# Combined Multistage Linear Genomic Selection Indices To Predict the Net Genetic Merit in Plant Breeding

**DOI:** 10.1534/g3.120.401171

**Published:** 2020-04-20

**Authors:** J. Jesus Cerón-Rojas, Jose Crossa

**Affiliations:** * Biometrics and Statistics Unit, International Maize and Wheat Improvement Center (CIMMYT), Apdo. Postal 6-641, 06600, México City, México and; ^†^Colegio de Postgraduados (COLPOS), CP56230, Montecillos, Edo. de Mexico, México

**Keywords:** Genomic estimated breeding value, Molecular marker effects, Multistage selection, Total selection response, Genomic Prediction GenPred, Shared Data Resources

## Abstract

A combined multistage linear genomic selection index (CMLGSI) is a linear combination of phenotypic and genomic estimated breeding values useful for predicting the individual net genetic merit, which in turn is a linear combination of the true unobservable breeding values of the traits weighted by their respective economic values. The CMLGSI is a cost-saving strategy for improving multiple traits because the breeder does not need to measure all traits at each stage. The *optimum* (OCMLGSI) and *decorrelated* (DCMLGSI) indices are the main CMLGSIs. Whereas the OCMLGSI takes into consideration the index correlation values among stages, the DCMLGSI imposes the restriction that the index correlation values among stages be zero. Using real and simulated datasets, we compared the efficiency of both indices in a two-stage context. The criteria we applied to compare the efficiency of both indices were that the total selection response of each index must be lower than or equal to the single-stage combined linear genomic selection index (CLGSI) response and that the correlation of each index with the net genetic merit should be maximum. Using four different total proportions for the real dataset, the estimated total OCMLGSI and DCMLGSI responses explained 97.5% and 90%, respectively, of the estimated single-stage CLGSI selection response. In addition, at stage two, the estimated correlations of the OCMLGSI and the DCMLGSI with the net genetic merit were 0.84 and 0.63, respectively. We found similar results for the simulated datasets. Thus, we recommend using the OCMLGSI when performing multistage selection.

The linear selection index can be a linear combination of phenotypic values (Smith 1936; Hazel and Lush 1942; Hazel 1943), genomic estimated breeding values (GEBV) (Ceron-Rojas *et al.* 2015; Cerón-Rojas and Crossa 2019), or of phenotypic values and GEBV (Dekkers 2007) jointly. In addition, it can also be a linear combination of phenotypic values and marker scores (Lande and Thompson 1990). All of these linear selection indices can be single-stage or multistage and are useful for selecting parents for the next generation and for predicting the individual net genetic merit, which, in turn, is a linear combination of the true unobservable breeding values of traits weighted by their respective economic values. The main aims of linear indices are to (1) predict the net genetic merit values of the candidates for selection, (2) maximize the selection response, and (3) provide the breeder with an objective rule for evaluating and selecting several traits simultaneously.

The selection response and the correlation between the index and the net genetic merit are the main index parameters; they are also the criteria used to compare the efficiency of any linear index to predict the net genetic merit. When the mean of the original population is zero, the selection response is the expected net genetic merit of the selected individuals (Smith 1936; Cochran 1951; Cerón-Rojas and Crossa 2018; Chapter 2). Both parameters give breeders an objective basis on which to validate the success of the adopted selection method.

Smith (1936) was the first to describe the single-stage linear phenotypic selection index (LPSI) theory under two assumptions: that the genotypic values that make up the net genetic merit are composed entirely of the additive effects of genes, and that the LPSI and the net genetic merit have bivariate normal distribution. When the phenotypic and genotypic covariance matrices of the individual traits under selection are known, the LPSI is the best linear predictor of the net genetic merit and is called optimum LPSI. The major advantages of the LPSI are that it assigns higher weights to traits whose differences are genetic and that it is relatively simple to analyze. Its disadvantages are that it requires large amounts of information, that the economic weights are difficult to assign and that the sampling error could be large. All linear selection indices associated with the LPSI theory have the same advantages and disadvantages (Cerón-Rojas and Crossa 2018, Chapters 2 to 6).

Cochran (1951) was the first to propose a two-stage LPSI, and Young (1964) generalized the Cochran (1951) approach to the multistage LPSI context. Young (1964) combined the independent culling selection method (Hazel and Lush 1942) and the LPSI theory to develop the optimum multistage LPSI (OMLPSI) for selecting several traits. One stage is the individual age (the length of time that a plant or animal has lived) at which the breeder measures and selects the individual based on the traits of interest into one specific selection cycle. Thus, suppose two vectors of individual traits, **x** and **y**, become evident at different animal or plant ages. In the single-stage context, both vectors of information are selected jointly using LPSI, whereas in the two-stage selection context, first **x** is selected at stage 1, and, at stage 2, we select **x** and **y** using OMLPSI. Young (1964) called the OMLPSI the “*part and whole index selection method*”, whereas Xu and Muir (1992) called it “*selection index updating*” because as traits become available, each subsequent index contains all traits available up to that stage.

Breeders apply the OMLPSI mainly in animal and tree breeding, where the target traits become evident at different individual ages. The OMLPSI is a cost-saving strategy for improving multiple traits because the breeder does not need to measure all traits at each stage. That is, the main advantage of the OMLPSI over the single-stage LPSI is that the breeder does not need to carry a large population of individuals throughout the multi-trait selection process. If the breeder selects with the LPSI, the same individuals for each trait of interest must be measured, and then the number of traits measured per mature individual is the same as for an immature individual. When traits have a developmental sequence in ontogeny, or when there are large differences in the costs of measuring several traits, the efficiency of OMLPSI over LPSI, in terms of cost saving, can be substantial (Xu and Muir 1991, 1992; Xu *et al.* 1995; Xie *et al.* 1997).

Some problems associated with the OMLPSI are as follow. First, after the first selection stage, the OMLPSI values could be non-normally distributed. Second, for more than two stages, the OMLPSI requires multiple integration techniques to derive selection intensities. Third, there are problems of convergence when the traits and the index values of successive stages are highly correlated, and finally, the computational time could be unacceptable if the number of selection stages becomes too high (Börner and Reinsch 2012). For these reasons, Xu and Muir (1992) developed s*election index updating* or the *decorrelated* multistage linear phenotypic selection index (DMLPSI).

In a similar manner as Cochran (1951) and Young (1964) developed the OMLPSI, Xu and Muir (1992) developed the DMLPSI combining the independent culling selection method and the LPSI theory for selecting several traits in the multistage context. However, while the OMLPSI theory takes into account the correlation among the OMLPSI values at different stages when predicting the net genetic merit, the DMLPSI theory imposes the restriction that the correlation between the DMLPSI values at different stages be zero; hence the name *decorrelated multistage index* (Börner and Reinsch 2012). Under this restriction, exact truncation points and selection intensities can be determined for a fixed selection proportion before selection is carried out, and the selected individual index values after the first selection stage could be normally distributed; in addition, it is not necessary to use multiple integration techniques to derive selection intensities. Xu and Muir (1992) derived a set of nonlinear equations in the DMLPSI context to obtain truncation points and selection intensities, and indicated that these equations may be solved iteratively using a multidimensional Newton method. Xu *et al.* (1995), however, found that the Newton method is sensitive to the initial values and frequently converges with solutions at a local maximum. Another problem associated with DMLPSI is that its selection responses and correlation with the net genetic merit are lower than the OMLPSI selection response and accuracy after the first selection stage (Börner and Reinsch 2012; Cerón-Rojas *et al.* 2019a, b).

In the marker-assisted selection (MAS) context, Lande and Thompson (1990) proposed a linear marker selection index (LMSI) which uses phenotypic and marker score values jointly to predict the net genetic merit. This index exploits the linkage disequilibrium between markers and quantitative trait loci (QTL) that occurs when inbred lines are crossed (Lange and Whittaker 2001). The LMSI requires regressing phenotypic values on marker coded values and, with these estimates, it constructs the marker score for each individual trait of the candidate for selection. Subsequently, the marker scores are combined with phenotypic information using the LMSI to predict the net genetic merit. Lande and Thompson (1990) assumed that the average effects on phenotype and the favorable alleles are known; however, this assumption is valid for major gene traits but not for quantitative traits that are affected by the environment, as well as many QTL with small effects that could interact with the environment and among themselves (Heffner *et al.* 2009). Several authors (Lange and Whittaker 2001; Meuwissen *et al.* 2001; Dekkers 2007) have criticized the LMSI approach because it makes inefficient use of the available data, as one would rather use all the available data in a single step to achieve maximally accurate estimates of marker effects. In addition, because the LMSI is based on only a few large QTL effects, it violates the selection index assumptions of multivariate normality and small changes in allele frequencies (Heffner *et al.* 2009).

Dekkers (2007) proposed a slightly modified version of the Lande and Thompson (1990) index. Instead of using marker scores, the Dekkers (2007) index uses the genomic estimated breeding values (GEBV) jointly with the phenotypic values to predict the net genetic merit. Céron-Rojas and Crossa (2018, Chapter 5) called this index the combined linear genomic selection index (CLGSI), and because it uses GEBV instead of marker scores, it is free of the problems (indicated earlier) that the LMSI presents. In the CLGSI context, all marker effects and GEBV of the genotyped individuals in the training population are estimated using marker and phenotypic data, and then the GEBVs are combined with the phenotypic values in a CLGSI to predict the net genetic merit and select parents for the next generation.

Xie and Xu (1998) extended the DMLPSI to the MAS context for developing a decorrelated multistage LMSI similar to the index of Lande and Thompson (1990). That is, the Xie and Xu (1998) index is an LMSI used to predict the net genetic merit in the multistage selection context. The main objective of Xie and Xu (1998) was to increase the efficiency of MAS in a two-stage breeding selection scheme. For this reason, they decided to select immature individuals (embryos) or seedlings at stage one based on a linear combination of trait molecular scores only, and, at stage two, to select mature individual traits based on a linear combination of trait molecular scores and phenotypic values jointly. According to Xie and Xu (1998), this selection method was implied in the paper by Lande and Thompson (1990) and the problem of these last two authors was how to find the selection intensities associated with a two-stage breeding scheme. For this reason, Xie and Xu (1998) adapted the DMLPSI theory to the MAS breeding context. This approach, however, has the same problems as those associated with the LMSI, which we indicated earlier.

In this work, we adapted the Dekkers (2007) index (which is an optimum index) to the multistage selection context. This index uses GEBV instead of marker scores; thus, it is free of the problems associated with the Xie and Xu (1998) index. We applied the proposed index in the two-stage context as follows. In stage one, we selected immature seedlings and embryos based on a linear combination of GEBV only, and, in stage two, we selected individual traits based on a linear combination of GEBV and phenotypic values jointly. This is the Xie and Xu (1998) idea but in the genomic selection context.

We validated the results of the proposed index using the optimum and decorrelated selection index theory in a two-stage breeding selection scheme (this approach can be extended to any number of stages). The optimum index was named *optimum combined multistage linear genomic selection index* (OCMLGSI), while the decorrelated index was called *decorrelated combined multistage linear genomic selection index* (DCMLGSI) because, at stage two, both indices use GEBV and phenotypic information jointly to predict the net genetic merit. While the OCMLGSI was based on the Dekkers (2007) and Young (1964) index theory, the DCMLGSI was based on the Xie and Xu (1998) and Xu and Muir (1992) index theory. We obtained the theoretical results of both indices under the assumption that the indices and the net genetic merit values have bivariate normal distribution. Under this assumption, the regression of the net genetic merit on any linear function of the phenotypic or GEBV values is linear (Kempthorne and Nordskog 1959) and the total selection response for two or more stages is the sum of each response obtained at each stage (Cochran 1951; Young 1964; Cerón-Rojas *et al.* 2019a).

We compared the relative efficiency of OCMLGSI and DCMLGSI using real and simulated datasets. The criteria used to compare the relative efficiency of both indices were that the total selection response of each index must be lower than, or equal to, the single-stage CLGSI (Dekkers 2007) selection response (Young 1964; Saxton 1983) and that the correlation of each index with the net genetic merit should be the maximum possible. The results of this study are the first ones comparing (with real and simulated data) the relative efficiency of the OCMLGSI with DCMLGSI efficiency using the total selection response and the maximized correlation with the net genetic merit as the main criteria to compare the efficiency of both indices.

## Material and Methods

We completed this section with three supplementary materials (Supplementary material 1, 2 and 3) that are located at http://hdl.handle.net/11529/10548356.

### Objectives of the combined multistage linear selection indices

Two objectives of the OCMLGSI and DCMLGSI are to maximize the selection response and predict the net genetic merit (H=w’g, where $w’=\left[\begin{array}{cc} {\text{w}}_{1} & {\text{w}}_{2} \end{array}\begin{array}{cc} \ldots & {\text{w}}_{t} \end{array}\right]$ and $g^{’}=\left[\begin{array}{cc} {\text{g}}_{1} & {\text{g}}_{2} \end{array}\begin{array}{cc} \ldots & {\text{g}}_{t} \end{array}\right]$ are vectors of economic weights and true unobservable breeding values, respectively, and t= number of traits). Additional OCMLGSI and DCMLGSI objectives are to select individuals with the highest H values as parents of the next generation, and provide the breeder with a rule for evaluating and selecting several traits simultaneously.

### The OCMLGSI and DCMLGSI at stage one

At stage 1, the OCMLGSI and DCMLGSI use only GEBV to predict the net genetic merit and select individual traits. The index to predict the individual net genetic merit at stage 1 is

$$I_{G}=w_{1}z_{1}+w_{2}z_{2}+\ldots +w_{t}z_{t}=w^{'}z,$$ (1)

where t= number of traits, $w’=\left[\begin{array}{cc} {\text{w}}_{1} & {\text{w}}_{2} \end{array}\begin{array}{cc} \ldots & {\text{w}}_{t} \end{array}\right]$ and $z^{\prime }=[\begin{array}{cccc} z_{1} & z_{2} & \cdots & z_{t} \end{array}]$ (Appendix 1, Equation A1 for details) are vectors of economic weights and genomic breeding values, respectively. At stage 1, Equation (1) is the same for both indices.

### The OCMLGSI and DCMLGSI parameters at stage one

The maximized OCMLGSI and DCMLGSI selection responses are

$$R_{O1}=k_{O1}\sqrt{w^{\prime }\Gamma w}\;\mathrm{and}\;R_{\;D1}=k_{D1}\sqrt{w^{\prime }\Gamma w},$$ (2)

respectively, where $k_{O1}$ and $k_{D1}$ (the selection intensities of each index) are the only difference between $R_{O1}$ and $R_{D1}$. In Equation (2), Γ=Var(z) is the covariance matrix of genomic breeding values (Appendix 1, Equation A2 for details), whereas w was defined earlier. The maximized correlation between $H=w^{\prime }g$ and $I=w^{'}z$ is $\rho_{{}_{HI}}=\frac{\sqrt{w^{\prime }\Gamma w}}{\sqrt{w\prime Cw}}$, where $\sqrt{w^{\prime }\Gamma w}$ is the standard deviation of the variance of I=wz, $\sigma_{H}=\sqrt{w^{\prime }Cw}$ is the standard deviation of the variance of H=w′g, and C=Var(g) is the covariance matrix of **g**.

### The combined multistage linear selection index and the net genetic merit at stage two

At stage 2, the OCMLGSI and DCMLGSI use genomic and phenotypic information jointly to predict the individual net genetic merit and can be written as

$$I_{C}={\beta^{\prime }}_{G}z+{\beta^{\prime }}_{y}y=\left[\begin{array}{cc} {\beta^{\prime }}_{G} & {\beta^{\prime }}_{y} \end{array}\right]\left[\begin{array}{c} z\\ y \end{array}\right]=\beta^{\prime }t,$$ (3)

where $y^{\prime }=[\begin{array}{cccc} y_{1} & y_{2} & ... & y_{t} \end{array}]$ is a vector of trait (t= number of traits) phenotypic values, and z was defined earlier; $\beta_{G}$ and $\beta_{y}$ are vectors of coefficients of genomic and phenotypic weight values, respectively; $\beta^{\prime }=[\begin{array}{cc} {\beta^{\prime }}_{G} & {\beta^{\prime }}_{y} \end{array}]$ and $t^{\prime }=[\begin{array}{cc} z^{\prime } & y^{\prime } \end{array}]$. The only difference between OCMLGSI and DCMLGSI is the way vector $\beta^{\prime }=[\begin{array}{cc} {\beta^{\prime }}_{G} & {\beta^{\prime }}_{y} \end{array}]$ is obtained.

The net genetic merit (H=w′g) can be written as

$$H={w^{\prime }}_{0}z+w^{\prime }g=[\begin{array}{cc} {w^{\prime }}_{0} & w^{\prime } \end{array}]\left[\begin{array}{c} z\\ g \end{array}\right]=a^{\prime }f,$$ (4)

where g, w and z have been defined above; ${w^{\prime }}_{0}=[\begin{array}{ccc} 0_{1} & \cdots & 0_{t} \end{array}]$ is a null vector associated with vector z, whereas $a^{\prime }=[\begin{array}{cc} {w^{\prime }}_{0} & w^{\prime } \end{array}]$ and $f^{\prime }=[\begin{array}{cc} z^{\prime } & g^{\prime } \end{array}]$.

### The OCMLGSI and DCMLGSI covariance matrices at stage two

Let P=Var(y), C=Var(g) and Γ=Var(z) be the phenotypic, genotypic and genomic covariance matrices, respectively; then

$$T=Var\left[\begin{array}{c} z\\ y \end{array}\right]=\left[\begin{array}{cc} \Gamma & \Gamma \\ \Gamma & P \end{array}\right]\;\mathrm{and}\;\Psi =Var\left[\begin{array}{c} z\\ g \end{array}\right]=\left[\begin{array}{cc} \Gamma & \Gamma \\ \Gamma & C \end{array}\right]$$ (5)

are block covariance matrices associated with OCMLGSI and DCMLGSI at stage 2. Equation (5) indicates that the covariance matrix between g and z is Γ=Cov(z,g)(Appendix 1, Equation 2). In Appendix 1 (Equations A6 to A8) we describe how we estimated matrices Γ, P, C.

### The OCMLGSI parameters at stage two

In Supplementary material 1 (see http://hdl.handle.net/11529/10548356), we extend the OCMLGSI theory to the multistage context. Here we present only the main results for the two stages. Since the OCMLGSI theory is based on LPSI theory, the OCMLGSI vector of coefficients (β) that maximizes the OCMLGSI response (and the correlation between the OCMLGSI and the net genetic merit values) is

$$\beta^{\prime }=[\begin{array}{cc} {\beta^{\prime }}_{G} & {\beta^{\prime }}_{y} \end{array}]=a^{\prime }\Psi T^{-1},$$ (6)

where $\beta_{G}=[I-{\left(P-\Gamma \right)}^{-1}(C-\Gamma )]w$ and $\beta_{y}={\left(P-\Gamma \right)}^{-1}(C-\Gamma )w$. By Equation (6), the maximized OCMLGSI selection response is

$$R_{O2}=k_{O2}\sqrt{\beta^{\prime }T\beta }=k_{O2}\sqrt{w^{\prime }\Gamma w+w^{\prime }(C-\Gamma ){\left(P-\Gamma \right)}^{-1}(C-\Gamma )w},$$ (7)

where $k_{O2}$ is the OCMLGSI intensity at stage 2. The total selection response for stages 1 and 2 is $R_{Ot}=R_{O1}+R_{O2}$. The maximized correlation between H (Equation 4) and the OCMLGSI is

$$\rho_{HI_{C}}=\frac{\sqrt{\beta^{\prime }T\beta }}{\sqrt{a^{\prime }\Psi a}}.$$ (8)

In Appendix 1 (Equation A9), we indicated how to estimate $R_{O2}$. Additional details of the parameters associated with Equations (7) and (8) are given in Supplementary material 3 (see http://hdl.handle.net/11529/10548356).

### The DCMLGSI parameters at stage 2

In Supplementary material 2 (see http://hdl.handle.net/11529/10548356), we extended the DCMLGSI theory to the multistage context, and we showed that the DCMLGSI vector of coefficients at stage 2 is

b=Kβ, (9)

where K=[I−U], $U=T^{-1}S{\left[S^{\prime }T^{-1}S\right]}^{-1}S^{\prime }$ and $\beta^{\prime }=a^{\prime }\Psi T^{-1}$ (Equation 6), whereas I is an identity matrix of the same size as matrix T; S is the matrix of constraints (Supplementary material 2, Equation S8, see http://hdl.handle.net/11529/10548356) that makes the covariances of the DCMLGSI values among stages null. Matrix K transforms the OCMLGSI vector of coefficients into the DCMLGSI vector of coefficients and is the only difference between Equations (6) and (9). At stage 1, S=0 (no constraints), K=I and b=β, the OCMLGSI vector of coefficients.

The maximized DCMLGSI selection response for stage two is

$$R_{D2}=k_{D2}\sqrt{b^{\prime }Tb},$$ (10)

where $k_{D2}$ is the DCMLGSI selection intensity at stage 2. The total selection response for stages 1 and 2 is $R_{Dt}=R_{D1}+R_{D2}$. In Appendix 1 (Equation A10), we indicate how to estimate $R_{\mathrm{D2}}$.

The maximized correlation between H (Equation 4) and DCMLGSI for stage 2 is

$$\rho_{HI_{D}}=\frac{\sqrt{b^{\prime }Tb}}{\sqrt{a^{\prime }\Psi a}}.$$ (11)

Note that the only difference between Equations (10) and (7), and Equations (11) and (8) is the vector of coefficients of each index.

### The OCMLGSI and DCMLGSI selection intensities for stages 1 and 2

The OCMLGSI selection intensities for stages 1 and 2 ($k_{O1}$ and $k_{O2}$, respectively) are those values associated with the maximum value of $R_{Ot}=R_{O1}+R_{O2}$, which were obtained with the method described in Appendix 2 (Equations A11 and A12). We obtained the DCMLGSI selection intensities for stages 1 and 2 ($k_{D1}$ and $k_{D2}$, respectively) using the Xu and Muir (1992) method. The value of $k_{D1}$ and $k_{D2}$ should maximize the total DCMLGSI selection response $R_{Dt}=R_{D1}+R_{D2}$.

### The genomic estimated breeding values for the OCMLGSI and DCMLGSI

Several authors (Ceron-Rojas *et al.* 2015; Isik *et al.* 2017; Cerón-Rojas and Crossa 2018, 2019) have given detailed descriptions of how to obtain genomic estimated breeding values (GEBV) that are predictors of unobservable individual trait breeding values. In the OCMLGSI and DCMLGSI context, we fitted, in a statistical model, phenotypic and marker data from the training population to estimate all available marker effects and obtain the GEBV (Appendix 1, Equations A3 and A4 for additional details).

### Testing the OCMLGSI and DCMLGSI normality assumption

Several authors (Shapiro and Wilk 1965; Mohd-Razali and Bee-Wah 2011; Rani Das and Rahmatullah-Imon 2016) have given details of how to perform a normality test procedure on a dataset, and many statistical packages provide graphs and normality tests (Crawley 2015).

For the real dataset, we corroborated the OCMLGSI and DCMLGSI normality assumption at stage 2 using graphical methods (histograms and normal Quantile-Quantile plots) and analytical test procedures (the Shapiro-Wilk and Kolmogorov-Smirnov normality tests). The corroboration procedure was as follows. In a two-stage context, let $p=q_{1}q_{2}$ be the fixed total proportion retained, where $q_{1}$ and $q_{2}$ denote the proportion selected at stages 1 and 2, respectively, and let n be the size of the dataset at stage 1; then $nq_{1}$ will be the size of the selected individuals at stage 1. We used the $nq_{1}$ individual genotypes and traits at stage 2 to construct graphs and statistical tests to corroborate the OCMLGSI and DCMLGSI normality assumption.

### Criteria for comparing OCMLGSI efficiency *vs.* DCMLGSI efficiency

The criteria to compare OCMLGSI efficiency *vs.* DCMLGSI efficiency were that the total OCMLGSI and DCMLGSI selection responses ($R_{Ot}=R_{O1}+R_{O2}$ and $R_{Dt}=R_{D1}+R_{D2}$, respectively) should be lower than, or equal to, the single-stage CLGSI selection response (R) described by Dekkers (2007) and Céron-Rojas and Crossa (2018, Chapter 5). In addition, the maximized correlation between the net genetic merit and the OCMLGSI and DCMLGSI (Equations 9 and 11, respectively) should be a maximum at each stage. Thus, the greater the OCMLGSI and DCMLGSI correlation with the net genetic merit, the more effective they are at predicting the net genetic merit and the selection response at each stage.

### Real data

We used a real maize (*Zea mays* L.) F2 population with 247 genotypes, 195 markers and 4 phenotypic traits: grain yield (GY, t/ha), plant height (PHT, cm), ear height (EHT, cm), and anthesis days (AD, d) to compare OCMLGSI efficiency *vs.* DCMLGSI efficiency to predict the net genetic merit. Beyene *et al.* (2015) described this dataset and denoted it as JMpop1 DTMA Mexico optimum environment. We assumed that the breeding objective was to increase GY while decreasing PHT, EHT, and AD. The vector of economic weights for those traits was $w^{’}=[\begin{array}{ccc} 5 & -0.1 & \begin{array}{cc} -0.1 & -1 \end{array} \end{array}]$, while the total proportions ($p=q_{1}q_{2}$) of retained values were p= 0.05, 0.10, 0.20 and 0.30 for both indices.

The estimated matrices of P, C, and Γ for all four traits were

$$\hat{P}=\left[\begin{array}{cccc} 1.40 & 4.69 & 3.59 & 0.12\\ 4.69 & 130.57 & 74.25 & 0.8\\ 3.59 & 74.25 & 92.96 & -1.32\\ 0.12 & 0.8 & -1.32 & 1.44 \end{array}\right],\;\hat{C}=\left[\begin{array}{cccc} 0.94 & 3.76 & 3.25 & 0.29\\ 3.76 & 72.24 & 51.13 & 1.99\\ 3.25 & 51.13 & 43.23 & -0.50\\ 0.29 & 1.99 & -0.50 & 0.90 \end{array}\right],\;\text{and}$$

$\hat{\Gamma }=\left[\begin{array}{cccc} 0.86 & 5.34 & 3.53 & 1.26\\ 5.34 & 57.92 & 32.18 & 9.62\\ 3.53 & 32.18 & 58.06 & 1.13\\ 1.26 & 9.62 & 1.13 & 3.32 \end{array}\right]$, respectively. At stage 1 we used matrix $\hat{\Gamma }$ and the vector of economic weights w to estimate the OCMLGSI and DCMLGSI selection responses (**Appendix 1**, Equation A9 and A10, respectively). At stage 2 we used all three matrices ($\hat{P}$, $\hat{C}$, and $\hat{\Gamma }$) to estimate matrices T and Ψ (Equation 5) as $\hat{T}=\left[\begin{array}{cc} \hat{\Gamma } & \hat{\Gamma }\\ \hat{\Gamma } & \hat{P} \end{array}\right]$ and $\hat{\Psi }=\left[\begin{array}{cc} \hat{\Gamma } & \hat{\Gamma }\\ \hat{\Gamma } & \hat{C} \end{array}\right]$, respectively, from where we estimated the OCMLGSI and DCMLGSI selection responses (**Appendix 1**, Equations A9 and A10, respectively).

### Simulated datasets

The datasets were simulated with QU-GENE software (Podlich and Cooper 1998) by Ceron-Rojas *et al.* (2015) using 2500 molecular markers and 315 QTL for eight phenotypic selection cycles (C0 to C7), each with four traits ($T_{1}$, $T_{2}$, $T_{3}$, and $T_{4}$), 500 genotypes and 4 replicates of each genotype. The authors distributed the markers uniformly across 10 chromosomes and the QTL randomly across the 10 chromosomes to simulate maize (*Zea mays* L.) populations. A different number of QTL affected each of the four traits: 300, 100, 60, and 40, respectively. The common QTL affecting the traits generated genotypic correlations of -0.5, 0.4, 0.3, -0.3, -0.2, and 0.1 between $T_{1}$ and $T_{2}$, $T_{1}$ and $T_{3}$, $T_{1}$ and $T_{4}$, $T_{2}$ and $T_{3}$, $T_{2}$ and $T_{4}$, $T_{3}$ and $T_{4}$, respectively. The economic weights for $T_{1}$, $T_{2}$, $T_{3}$, and $T_{4}$ were 1, -1, 1, and 1, respectively, in all selection cycles. To illustrate the efficiency of the OCMLGSI and DCMLGSI to predict the net genetic merit, in this work we used six selection cycles (C1 to C6) with p=0.05, 0.10 and 0.20 in each cycle. We selected all four traits in each selection cycle.

### Data availability

The real and simulated datasets are available in the *Application of a Genomics Selection Index to Real and Simulated Data* repository, at http://hdl.handle.net/11529/10199. The real dataset used in this work is the folder named “File Real_Data_Sets_GSI” which contains four folders named “DATA_SET-3, 4, 5, and 6”. Each of the four folders contains four Excel data files. The four Excel data files within the folder DATA_SET-3 are as follows: DATA_SET-3_Markers_Cycle-0, 1, 2, and DATA_SET-3_Phenotypic_Cycle-0. The first three Excel files contain the marker-coded values for cycles 0, 1, and 2, while the Excel file DATA_SET-3_Phenotypic_Cycle-0 contains the phenotypic information of cycle 0 (training population). The Excel data files of the other folders were described in a similar manner as for folder 3. In this work, we used dataset 3 for cycle 0 to make selections and to estimate the selection response and the correlation of the OCMLGSI and DCMLGSI with the net genetic merit. The results are presented in Table 1.

**Table 1:** **Real data for different total proportions (**$p=q_{1}q_{2}$**) retained; estimated optimum and decorrelated combined multistage linear genomic selection index truncation points (**$u_{1}$ **and** $u_{2}$**), proportions retained (**$q_{1}$ **and** $q_{2}$**), selection intensities (**$k_{1}$ **and** $k_{2}$**) and maximized estimated selection responses (**${\overset{\text{\textasciicircum{}}}{R}}_{1}$, ${\overset{\text{\textasciicircum{}}}{R}}_{2}$ **and** ${\overset{\text{\textasciicircum{}}}{R}}_{t}={\overset{\text{\textasciicircum{}}}{R}}_{1}+{\overset{\text{\textasciicircum{}}}{R}}_{2}$**) for stages 1 and 2. Values of** $\overset{\text{\textasciicircum{}}}{R}$ **correspond to maximized estimated single-stage combined linear genomic selection index responses.**

Optimum index										
*p*	$u_{1}$	$u_{2}$	$q_{1}$	$q_{2}$	$k_{1}$	$k_{2}$	${\hat{R}}_{1}$	${\hat{R}}_{2}$	${\hat{R}}_{t}$	$\hat{R}$
0.05	0.61	0.90	0.27	0.18	1.22	1.44	3.52	4.66	8.18	8.41
0.10	0.31	0.63	0.38	0.26	1.01	1.24	2.90	4.07	6.97	7.16
0.20	−0.06	0.30	0.52	0.38	0.76	1.00	2.19	3.38	5.57	5.71
0.30	−0.34	0.06	0.63	0.47	0.60	0.84	1.72	2.91	4.62	4.73
Average	0.13	0.47	0.45	0.33	0.90	1.13	2.58	3.75	6.34	6.50
Decorrelated index										
*p*	$u_{1}$	$u_{2}$	$q_{1}$	$q_{2}$	$k_{1}$	$k_{2}$	${\hat{R}}_{1}$	${\hat{R}}_{2}$	${\hat{R}}_{t}$	$\hat{R}$
0.05	0.76	0.76	0.22	0.22	1.33	1.34	3.84	3.87	7.71	8.41
0.10	0.48	0.48	0.32	0.32	1.12	1.13	3.23	3.25	6.49	7.16
0.20	0.13	0.14	0.45	0.45	0.88	0.89	2.54	2.56	5.10	5.71
0.30	−0.12	−0.12	0.55	0.55	0.72	0.73	2.08	2.09	4.17	4.73
Average	0.31	0.32	0.38	0.38	1.02	1.02	2.92	2.94	5.87	6.50

Folder Simulated_Data_GSI contains two folders: Data_Phenotypes_April-26-15 and Haplotypes_GSI_April-26-15. In turn, folder Data_Phenotypes_April-26-15 also contains two folders: GSI_Phenotypes-05 and PSI_Phenotypes-05. Within folder GSI_Phenotypes-05, there are six Excel data files, each denoted as C2_GSI_05_Pheno, C3_GSI_05_Pheno, C4_GSI_05_Pheno, C5_GSI_05_Pheno and C6_GSI_05_Pheno, corresponding to the phenotypic simulated information for the genomic selection index for cycles 2-7. In addition, folder GSI_Phenotypes-05 contains eight Excel datasets denoted as C0_Pheno_05, C1_PSI_05_Pheno, C2_PSI_05_Pheno, C3_PSI_05_Pheno, C4_PSI_05_Pheno, C5_PSI_05_Pheno, C6_PSI_05_Pheno, and C7_PSI_05_Pheno corresponding to the phenotypic simulated information for the phenotypic selection index for cycles 0-7. File Haplotypes_GSI_April-26-15 contains the haplotypes of the markers for cycles 0-7 of GSI. We present the results of the simulated datasets in Tables 2 and 3 for cycles 1 to 6.

**Table 2:** **Simulated data for estimated optimum and decorrelated combined multistage linear genomic selection indices responses (**${\overset{\text{\textasciicircum{}}}{R}}_{1}$, ${\overset{\text{\textasciicircum{}}}{R}}_{2}$, ${\overset{\text{\textasciicircum{}}}{R}}_{t}={\overset{\text{\textasciicircum{}}}{R}}_{1}+{\overset{\text{\textasciicircum{}}}{R}}_{2}$**) and single-stage combined linear genomic selection index responses (**${\overset{\text{\textasciicircum{}}}{R}}_{0.05}$, ${\overset{\text{\textasciicircum{}}}{R}}_{0.10}$, ${\overset{\text{\textasciicircum{}}}{R}}_{0.20}$**) for six simulated selection cycles in a two-stage breeding scheme for total proportions retained** $p=q_{1}q_{2}=$**0.05, 0.10, and 0.20.**

	Optimum index												Single-stage index				
	p=0.05			p=0.10					p=0.20								
Cycle	${\hat{R}}_{1}$	${\hat{R}}_{2}$	${\hat{R}}_{t}$	${\hat{R}}_{1}$		${\hat{R}}_{2}$		${\hat{R}}_{t}$	${\hat{R}}_{1}$	${\hat{R}}_{2}$		${\hat{R}}_{t}$	${\hat{R}}_{0.05}$		${\hat{R}}_{0.10}$		${\hat{R}}_{0.20}$
1	11.76	9.05	20.81	9.77		7.97		17.73	7.49	6.70		14.19	21.32		18.14		14.47
2	12.67	6.06	18.73	10.57		5.40		15.97	8.17	4.62		12.79	19.06		16.22		12.94
3	13.39	4.03	17.42	11.21		3.66		14.87	8.70	3.22		11.92	17.58		14.95		11.93
4	10.73	6.34	17.07	8.94		5.61		14.55	6.89	4.76		11.65	17.43		14.83		11.83
5	9.57	6.62	16.19	7.96		5.84		13.80	6.11	4.93		11.04	16.57		14.09		11.24
6	9.44	5.15	14.59	7.87		4.57		12.44	6.07	3.89		9.96	14.88		12.66		10.10
Average	11.26	6.21	17.47	9.39		5.51		14.89	7.24	4.69		11.93	17.81		15.15		12.08
	Decorrelated index																
	p=0.05			p=0.10				p=0.20					Single-stage index				
Cycle	${\hat{R}}_{1}$	${\hat{R}}_{2}$	${\hat{R}}_{t}$	${\hat{R}}_{1}$	${\hat{R}}_{2}$		${\hat{R}}_{t}$	${\hat{R}}_{1}$		${\hat{R}}_{2}$	${\hat{R}}_{t}$		${\hat{R}}_{0.05}$	${\hat{R}}_{0.10}$		${\hat{R}}_{0.20}$	
1	12.84	6.77	19.61	10.91	5.61		16.52	8.70		4.30	13.00		21.32	18.14		14.47	
2	13.84	3.86	17.70	11.81	3.13		14.94	9.48		2.30	11.79		19.06	16.22		12.94	
3	14.62	1.94	16.55	12.50	1.51		14.01	10.06		1.03	11.10		17.58	14.95		11.93	
4	11.72	4.39	16.10	9.99	3.59		13.58	8.00		2.70	10.70		17.43	14.83		11.83	
5	10.45	4.81	15.26	8.89	3.97		12.86	7.10		3.02	10.12		16.57	14.09		11.24	
6	10.31	3.47	13.77	8.79	2.82		11.62	7.05		2.11	9.16		14.88	12.66		10.10	
Average	12.29	4.21	16.50	10.48	3.44		13.92	8.40		2.58	10.98		17.81	15.15		12.08	

**Table 3:** **Simulated data for estimated maximum correlation values of optimum (**${\overset{\text{\textasciicircum{}}}{\rho }}_{1}$ **and** ${\overset{\text{\textasciicircum{}}}{\rho }}_{2}$**) and decorrelated (**${\overset{\text{\textasciicircum{}}}{r}}_{1}$ **and** ${\overset{\text{\textasciicircum{}}}{r}}_{2}$**) combined multistage linear genomic selection indices with the net genetic merit under a two-stage (each stage denoted by 1 and 2) breeding scheme for six simulated cycles.**

	Optimum index		Decorrelated index	
Cycle	${\hat{\rho }}_{1}$	${\hat{\rho }}_{2}$	${\hat{r}}_{1}$	${\hat{r}}_{2}$
1	0.73	0.86	0.73	0.56
2	0.78	0.82	0.78	0.47
3	0.83	0.78	0.83	0.38
4	0.74	0.80	0.74	0.50
5	0.71	0.80	0.71	0.52
6	0.73	0.76	0.73	0.47
Average	0.75	0.80	0.75	0.48

### Matching phenotypic and genomic real data

To estimate the OCMLGSI and DCMLGSI parameters and make selections, we use the following two Excel files: (1) “*DATA_SET-3_Phenotypic_Cycle-0*” (which contains the raw phenotypic data) and (2) the “*DATA_SET-3_Markers_Cycle-0*” (which contains the coded molecular markers). Both datasets are in the folder named “***DATA_SET-3***”.

### Matching phenotypic and genomic simulated data

To estimate the OCMLGSI and DCMLGSI parameters and to make selections, we used the data of two folders. The first one is called “PSI_Phenotypes-05” (which contains the raw phenotypic data of six Excel files named: C1_PSI_05_Pheno to C6_PSI_05_Pheno) and a second one named “Haplotypes_GSI_April-26-15”(which contains the raw marker data of six text files named: C1_PSI_S2_05_Haplo.pop to C6_PSI_S2_05_Haplo.pop). Both datasets are in the folder named “***Simulated_Data_GSI***”. To estimate the OCMLGSI and DCMLGSI parameters, the foregoing files were matched as follows. For selection cycle 1, we matched the Excel file C1_PSI_05_Pheno with the text files C1_PSI_S2_05_Haplo.pop; for selection cycle 2, we matched the Excel file C2_PSI_05_Pheno with the text files C2_PSI_S2_05_Haplo.pop, etc. Finally, in cycle 6, we matched the Excel file C6_PSI_05_Pheno with the text files C6_PSI_S2_05_Haplo.pop.

## Results

### Real data

#### Estimated OCMLGSI parameters for stages 1 and 2:

The estimated OCMLGSI values at stages 1 and 2 were ${\hat{I}}_{1}=w^{\prime }\hat{z}$ and ${\hat{I}}_{2}={\hat{\beta }}^{\prime }t$, respectively, where $w^{\prime }$ and ${\hat{\beta }}^{\prime }$ were the estimated vector or coefficient (Appendix 1, Equation A9), $\hat{z}$ (Appendix 1, Equations A3 and A4) was a vector of GEBV associated with the vector of traits y, and $t^{\prime }=[\begin{array}{cc} \hat{z^{\prime }} & y^{\prime } \end{array}]$. The maximum estimated total OCMLGSI selection response was ${\hat{R}}_{Ot}=k_{O1}\sqrt{w^{\prime }\hat{\Gamma }w}+k_{O2}\sqrt{\hat{\beta^{\prime }}{\hat{T}}^{*}\hat{\beta }}$, where ${\hat{R}}_{O1}=k_{O1}\sqrt{w^{\prime }\hat{\Gamma }w}$ and ${\hat{R}}_{O2}=k_{O2}\sqrt{\hat{\beta^{\prime }}{\hat{T}}^{*}\hat{\beta }}$ were the estimated selection responses at each stage, and matrix ${\hat{T}}^{*}$ was the adjusted matrix $\hat{T}$ for prior selection on ${\hat{I}}_{1}$ (Appendix 3, Equation A13).

**Figure 1:**
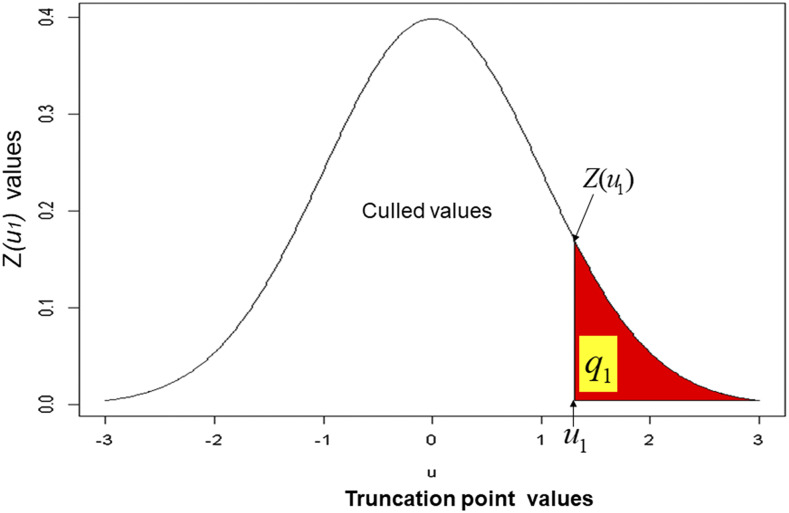
Theoretical relationship between one truncation point ($u_{1}$) values, the total proportion retained ($q_{1}$) and the density values ($z(u_{1})$) of the truncation point.

**Figure 2:**
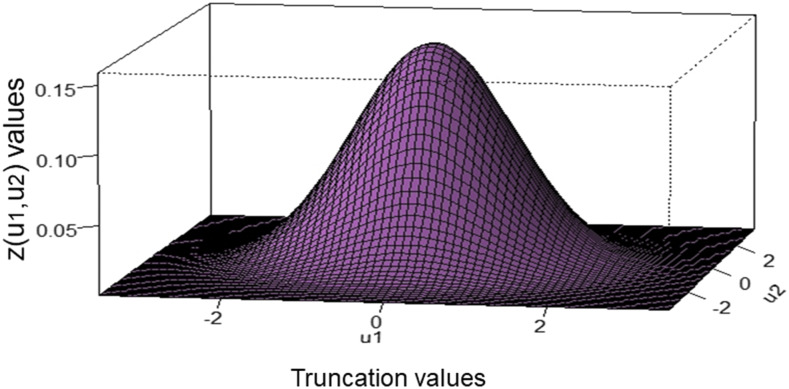
Theoretical relationship between two truncation point (u1 and u2) values and the density values [z(u1, u2)] of the truncation points.

**Figure 3:**
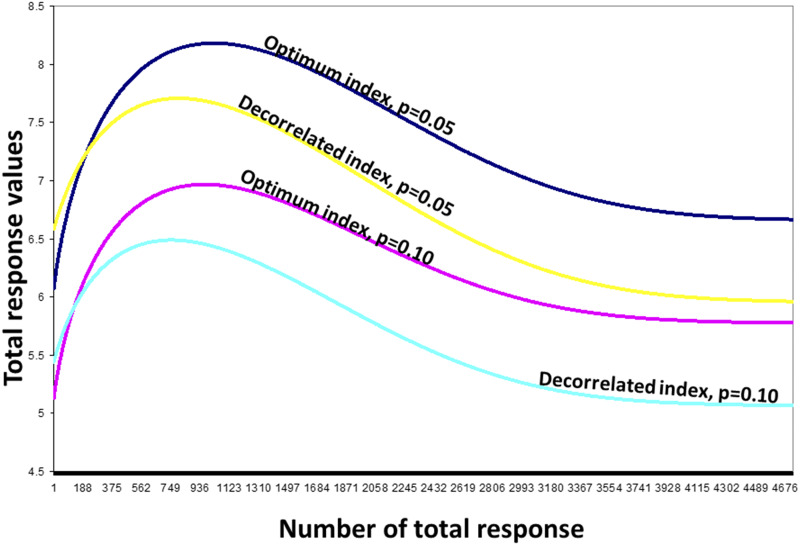
Distribution of the total estimated OCMLGSI and DCMLGSI selection response values, under a two-stage breeding scheme, for a real dataset with $p=q_{1}q_{2}=$0.05 and 0.10.

Figure 1 shows the theoretical relationship between one truncation point ($u_{1}$) value, the proportion retained ($q_{1}$), and the density values ($z(u_{1})=e^{-0.5u_{1}^{2}}/\sqrt{2\pi }$) of the truncation point at stage 1, while Figure 2 describes the theoretical relationship between two truncation point ($u_{1}$ and $u_{2}$) values and their density values [$z(u_{1},u_{2})$, Appendix 2, Equation A11] for two stages. In Appendix 2 (Equation A12), we described a method to obtain the OCMLGSI selection intensities ($k_{O1}$ and $k_{O2}$) that maximize ${\hat{R}}_{Ot}$ for both stages. We found the $k_{O1}$ and $k_{O2}$ values as follows: for a fixed value $p=q_{1}q_{2}$, we used an iterative process with an R-code (Kabakoff 2011) where, by successively changing the possible values of $q_{1}$ ($q_{2}=p/q_{1}$), $u_{1}$, and $u_{2}$, we found the maximum value of the estimated total OCMLGSI selection response ${\hat{R}}_{Ot}$ (Figure 3). Thus, for p=0.05, the values of the truncation points ($u_{1}=0.61$ and $u_{2}=0.90$), proportions retained ($q_{1}=0.27$ and $q_{2}=0.18$) and selection intensities ($k_{O1}=1.22$ and $k_{O2}=1.44$) at both stages, were those associated with the maximum ${\hat{R}}_{Ot}=8.18$ value (Figure 3).

In the OCMLGSI and DCMLGSI context, $p=q_{1}q_{2}$, $q_{2}=\frac{p}{q_{1}}$, that is, the only parameter that changes is $q_{1}$ because p is fixed. The same is true for the truncation points ($u_{1}$ and $u_{2}$) because $u_{1}=\Phi^{-1}(1-q_{1})$ and $u_{2}=\Phi^{-1}(1-q_{2})$, where $\Phi^{-1}()$is the inverse function of the standard normal distribution (Xu and Muir 1991, 1992). Thus, in this context, $k_{O1}$, $k_{O2}$, and ${\hat{R}}_{Ot}$ values are mainly associated with the possible values of $q_{1}$.

Figure 3 presents the ${\hat{R}}_{Ot}$ values on the *y*-axis, whereas the *x*-axis presents the possible values of the combinations of $k_{O1}$ and $k_{O2}$ for all possible realizations of ${\hat{R}}_{Ot}$, and for this reason, the *x*-axis takes values from 1 to 4676. For *x*-axis number 1, we should have a possible combination of $k_{O1}$ and $k_{O2}$ values for a possible realization of ${\hat{R}}_{Ot}$, which we could denote as ${\hat{R}}_{Ot}(1)$, while for *x*-axis number 4676, we should have an additional realization denoted as ${\hat{R}}_{Ot}(4676)$. The ${\hat{R}}_{Ot}(1)$ and ${\hat{R}}_{Ot}(4676)$ values are different and their values should appear in the graph and on the *y*-axis. The ${\hat{R}}_{Ot}=8.18$ value was between ${\hat{R}}_{Ot}(1)$ and ${\hat{R}}_{Ot}(4676)$.

In the single-stage context, when p=0.05, k=2.06, and the estimated CLGSI selection response was $\hat{R}=8.41$, which should be higher than, or equal to ${\hat{R}}_{Ot}=8.18$ (Young 1964; Saxton 1983). In this case, ${\hat{R}}_{Ot}=8.18$ explained 97.27% of the $\hat{R}=8.41$ value. Thus, both selection responses were similar. In addition, because matrix ${\hat{T}}^{*}$ had more information than matrices $\hat{\Gamma }$ and $\hat{P}$ (Appendix 1, Equations A5 to A9), ${\hat{R}}_{Ot}=8.18$ was higher than the estimated LGSI and LPSI selection responses in the single-stage context (${\hat{R}}_{G}=k\sqrt{w^{\prime }\hat{\Gamma }w}=5.93$ and ${\hat{R}}_{P}=k\sqrt{\hat{\delta^{\prime }}\hat{P}\hat{\delta }}=6.77$, respectively), for p=0.05, and, in addition, ${\hat{R}}_{Ot}\neq {\hat{R}}_{G}+{\hat{R}}_{P}$ (see Supplementary material 3 for details).

For $p=q_{1}q_{2}=$0.10, 0.20, and 0.30, the ${\hat{R}}_{Ot}$ values were 6.97, 5.57, and 4.62, whereas the $\hat{R}$ values were 7.16, 5.71, and 4.73, respectively (Table 1). Thus, the ${\hat{R}}_{Ot}$ values explained 97.35, 97.55, and 97.68%, respectively, of the $\hat{R}$ values. That is, the estimated selection responses of OCMLGSI and CLGSI were very similar.

The estimated maximized OCMLGSI correlations with the net genetic merit (Equation 8) at stages 1 and 2 were ${\hat{\rho }}_{1}=\frac{\sqrt{w^{\prime }\hat{\Gamma }w}}{\sqrt{w^{\prime }\hat{C}w}}=0.63$ and ${\hat{\rho }}_{2}=\frac{\sqrt{{\hat{\beta }}^{\prime }{\hat{T}}^{*}\hat{\beta }}}{\sqrt{a^{\prime }{\hat{\Psi }}^{*}a}}=0.84$, respectively, where $\sqrt{w^{\prime }\hat{\Gamma }w}$ and $\sqrt{{\hat{\beta }}^{\prime }{\hat{T}}^{*}\hat{\beta }}$ were the standard deviations of the variances of ${\hat{I}}_{1}$ and ${\hat{I}}_{2}$, whereas $\sqrt{w^{\prime }\hat{C}w}$ and $\sqrt{a^{\prime }{\hat{\Psi }}^{*}a}$ were the estimated standard deviations of the net genetic merit (Equation 4) at each stage. Matrix ${\hat{\Psi }}^{*}$ was the adjusted matrix $\hat{\Psi }$ for prior selection on ${\hat{I}}_{1}$ (**Appendix 3**, Equation A14) and because matrices ${\hat{T}}^{*}$ and ${\hat{\Psi }}^{*}$ had more information than matrices $\hat{C}$ and $\hat{\Gamma }$, ${\hat{\rho }}_{2}>{\hat{\rho }}_{1}$.

### Estimated DCMLGSI parameters for stages 1 and 2

The estimated DCMLGSI values for both stages were ${\hat{I}}_{D1}=w^{\prime }\hat{z}$ and ${\hat{I}}_{D2}={\hat{b}}^{\prime }t$, where $w^{\prime }$was the vector of economic weights and ${\hat{b}}^{\prime }$ was the estimated vector of coefficients, whereas $\hat{z}$, $t^{\prime }=[\begin{array}{cc} \hat{z^{\prime }} & y^{\prime } \end{array}]$ and y were described earlier. For $p=q_{1}q_{2}=0.05$, the DCMLGSI values of the truncation points ($u_{1}=0.76$ and $u_{2}=0.76$), proportions retained ($q_{1}=0.22$ and $q_{2}=0.22$) and selection intensities ($k_{D1}=1.33$ and $k_{D2}=1.44$) maximized ${\hat{R}}_{Dt}=k_{D1}\sqrt{w^{\prime }\hat{\Gamma }w}+k_{D2}\sqrt{{\hat{b}}^{\prime }\hat{T}\hat{b}}=7.71$ (Figure 3) and were obtained with the Xu and Muir (1992) method. In this case, ${\hat{R}}_{Dt}=7.71$ (Appendix 1, Equation A10) explained 91.68% of the estimated CLGSI selection response ($\hat{R}=8.41$) and was lower than the estimated total OCMLGSI selection response (${\hat{R}}_{Ot}=8.18$).

For $p=q_{1}q_{2}=$0.10, 0.20, and 0.30, the ${\hat{R}}_{Dt}$ values (6.49, 5.10, and 4.17, respectively) explained 90.64, 89.32, and 88.16%, respectively, of the $\hat{R}$ values (7.16, 5.71, and 4.73, respectively) (Table 1). Thus, for all p values, the estimated total OCMLGSI response was higher than the estimated total DCMLGSI response.

The estimated DCMLGSI correlations with the net genetic merit at stages 1 and 2 were ${\hat{r}}_{1}=\frac{\sqrt{w^{\prime }\hat{\Gamma }w}}{\sqrt{w^{\prime }\hat{C}w}}=0.63$ and ${\hat{r}}_{2}=\frac{\sqrt{{\hat{b}}^{\prime }\hat{T}\hat{b}}}{\sqrt{a^{\prime }\hat{\Psi }a}}=0.63$, respectively, where $\sqrt{w^{\prime }\hat{\Gamma }w}$ and $\sqrt{{\hat{b}}^{\prime }\hat{T}\hat{b}}$ were the standard deviations of the variances of ${\hat{I}}_{D1}$ and ${\hat{I}}_{D2}$, respectively, whereas $\sqrt{w^{\prime }\hat{C}w}$ and $\sqrt{a^{\prime }\hat{\Psi }a}$ were the estimated standard deviations of the variances of the net genetic merit (Equation 4). In this case, ${\hat{r}}_{1}={\hat{r}}_{2}$.

### Truncation points, proportion retained and selection intensities

Table 1 presents the OCMLGSI and DCMLGSI truncation points, proportions retained and selection intensities for $p=q_{1}q_{2}=$0.05, 0.10, 0.20, and 0.30 in a two-stage context. When the $p=q_{1}q_{2}$ values changed from 0.05 to 0.30, the truncation point values decreased, the proportions retained values increased and the selection intensity values decreased in both indices, as we would expect. In addition, while the DCMLGSI truncation points and proportions retained values were the same at both stages, the OCMLGSI truncation point values at stage 1 were lower than at stage 2, and then $q_{1}>q_{2}$. For this reason, the OCMLGSI selection intensity was different from the DCMLGSI selection intensity.

### Simulated data

#### Estimated maximized OCMLGSI and DCMLGSI selection responses:

For $p=q_{1}q_{2}=$0.05, 0.10, and 0.20, Table 2 presents the estimated maximized OCMLGSI and DCMLGSI selection responses (${\hat{R}}_{1}$, ${\hat{R}}_{2}$, ${\hat{R}}_{t}={\hat{R}}_{1}+{\hat{R}}_{2}$) and the estimated maximized single-stage CLGSI responses (${\hat{R}}_{0.01}$, ${\hat{R}}_{0.10}$, ${\hat{R}}_{0.20}$) for six simulated selection cycles in a two-stage breeding selection scheme. For p=0.05, the average of the estimated total OCMLGSI selection responses (17.47) explained 98.10% of the average of the estimated CLGSI selection responses (17.81), whereas for p= 0.10 and 0.20, the average of the estimated total OCMLGSI selection responses (14.89 and 11.93, respectively) explained 98.28% and 99.42% of the average of the CLGSI selection response (15.15 and 12.08, respectively). Thus, for this dataset, the OCMLGSI and CLGSI results were equivalent for all p values.

For p=0.05, the average of the estimated total DCMLGSI selection responses (16.50) explained 92.64% of the average of the CLGSI selection responses (17.81), whereas for p= 0.10 and 0.20, the average of the estimated total DCMLGSI selection responses (13.92 and 10.98, respectively) explained 91.88% and 90.89% of the average of the CLGSI selection responses (15.15 and 12.08, respectively).

The foregoing results indicate that while the average of the total OCMLGSI selection responses (for all p values) explained 98.60% of the average of the CLGSI, the average of the total DCMLGSI selection responses (for all pvalues) explained 91.80% of the average of the CLGSI. Thus, OCMLGSI accuracy was higher than DCMLGSI accuracy for predicting the selection response.

#### Estimated OCMLGSI and DCMLGSI correlations with the net genetic merit:

Table 3 presents the estimated and maximized values of the OCMLGSI (${\hat{\rho }}_{1}$ and ${\hat{\rho }}_{2}$) and DCMLGSI (${\hat{r}}_{1}$ and ${\hat{r}}_{2}$) correlations with the net genetic merit in a two-stage context for six simulated selection cycles. At stage 1, the averages of the estimated OCMLGSI and DCMLGSI correlations were the same. However, at stage 2, the average of the estimated OCMLGSI correlations with net genetic merit was 40.0% higher than the average of the estimated DCMLGSI correlations for six simulated selection cycles.

#### Histograms and quantile-quantile plots for the estimated OCMLGSI and DCMLGSI values at stage two:

**Figure 4:**
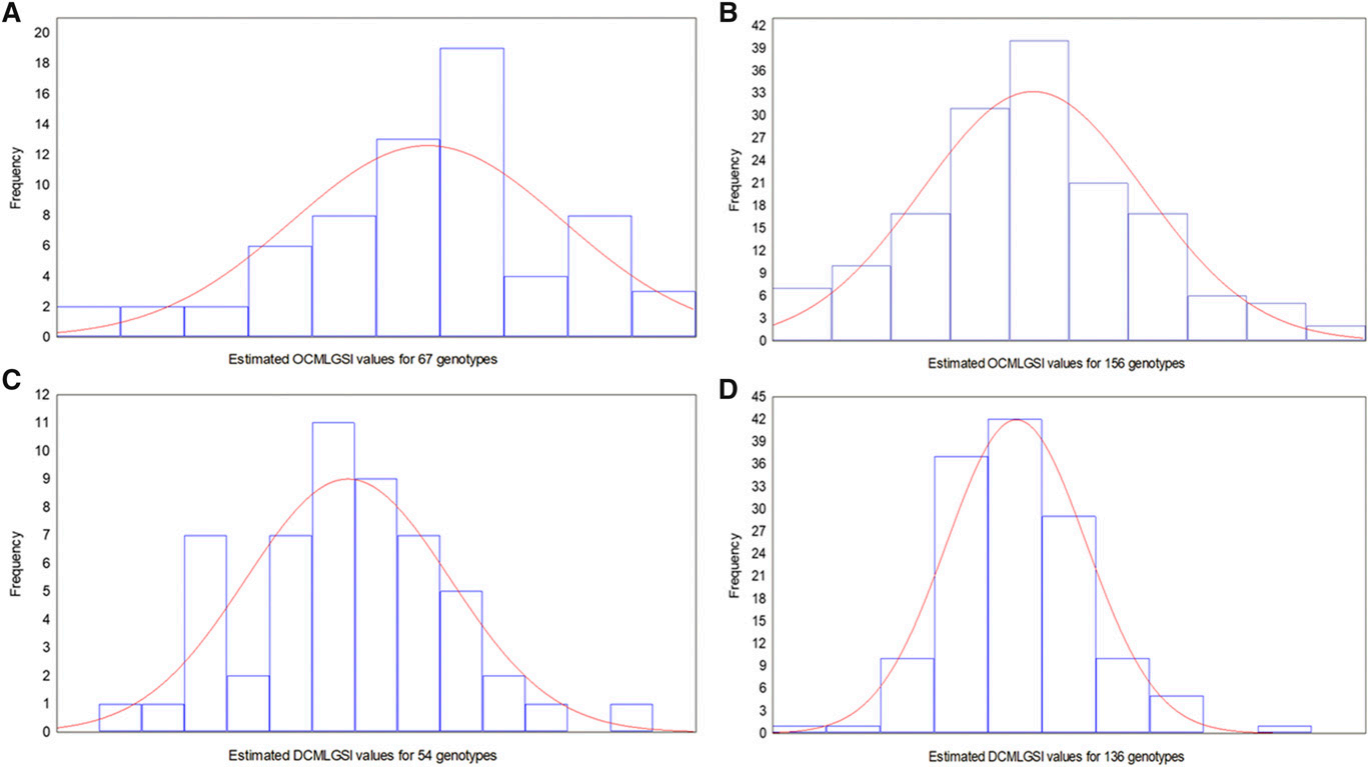
Histograms of the estimated OCMLGSI and DCMLGSI values at stage 2, for a real dataset, when the number of genotypes was 67 (A) and 156 (B) for OCMLGSI, and 54 (C) and 136 (D) for DCMLGSI.

**Figure 5:**
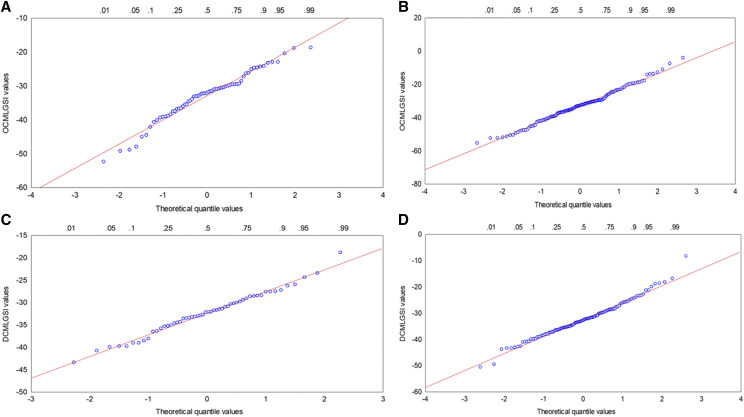
Quantile-quantile plot of the estimated OCMLGSI and DCMLGSI values at stage 2 for a real dataset when the number of genotypes was 67 (A) and 156 (B) for OCMLGSI, and 54 (C) and 136 (D) for DCMLGSI.

We used the real dataset selected in cycle 1 to test the normality assumption of the estimated OCMLGSI and DCMLGSI values at stage 2. For $p=q_{1}q_{2}=$ 0.05 and 0.30, the $q_{1}$ values for OCMLGSI were 0.27 and 0.63, while those values for DCMLGSI were 0.22 and 0.55, respectively. Then, at stage 2, (0.27)(247)=67 and (0.63)(247)=156 were the number of genotypes for OCMLGSI, whereas for DCMLGSI, the number of genotypes were (0.22)(247)=54 and (0.55)(247)=136, where 247 was the number of genotypes at stage 1. With these genotypes, we constructed histograms (Figure 4) and quantile–quantile plots (Figure 5) of the OCMLGSI and DCMLGSI values at stage 2. If the OCMLGSI and DCMLGSI values have normal distribution, the histograms of the values of both indices should not show a strong negative or positive skew in the OCMLGSI and DCMLGSI values seen in the histogram. Similarly, if the OCMLGSI and DCMLGSI values are normally distributed, then they should form a straight line in the quantile–quantile plots. If there are departures from normality, the OCMLGSI and DCMLGSI values should show up as various kinds of non-linearity, *e.g.*, S-shaped or banana-shaped in the quantile–quantile plots (Crawley 2015).

When the number of genotypes changed from 67 (Figure 4a) to 156 (Figure 4b), the estimated OCMLGSI values were closer to the normal distribution. The same was true for the estimated DCMLGSI values when the number of genotypes changed from 54 (Figure 4c) to 136 (Figure 4d). In addition, the quantile–quantile plots indicate that when the number of genotypes changed from 67 (Figure 5a) to 156 (Figure 5b), the estimated OCMLGSI values tended to form a straight line. The same was true for the estimated DCMLGSI values when the number of genotypes changed from 54 (Figure 5c) to 136 (Figure 5d).

#### Normality test for the estimated OCMLGSI and DCMLGSI values at stage two:

For the real dataset, we present the results of the Shapiro-Wilk and Kolmogorov-Smirnov normality tests of the estimated OCMLGSI and DCMLGSI values at stage 2 when the number of genotypes was 67 and 156 for OCMLGSI, and 54 and 136 for DCMLGSI. We tested the null hypothesis that the estimated OCMLGSI and DCMLGSI values have normal distribution. The statistical value of the Shapiro-Wilk test should be close to 1.0 to accept the null hypothesis, while the statistical value of the Kolmogorov-Smirnov test should be close to 0.0 to accept the null hypothesis (Rani Das and Rahmatullah-Imon 2016). In the present case, for the OCMLGSI values (67 and 156), the Shapiro-Wilk test values were 0.976 and 0.987, respectively, while the Kolmogorov-Smirnov test values were 0.088 and 0.089, respectively. Thus, the null hypothesis was true for the estimated OCMLGSI values. Similarly, for the DCMLGSI values (54 and 136), the Shapiro-Wilk test values were 0.988 and 0.984, respectively, while the Kolmogorov-Smirnov test values were 0.067 and 0.058, respectively. Thus, we accepted that the estimated DCMLGSI values approach the normal distribution.

## Discussion

### The DCMLGSI restrictions imposed on the covariance values

The DCMLGSI imposed the restriction that the covariance between DCMLGSI values among stages be zero. This restriction was to ensure the existence of solutions for the truncation points at different stages without resorting to numerical multiple integration (Xu and Muir 1992; Xie and Xu 1998). However, the restriction decreased the estimated DCMLGSI selection response and the estimated DCMLGSI correlation with the net genetic merit after stage 1. Xu and Muir (1992) indicated that the loss of DCMLGSI efficiency after stage 1 is justified because their method for obtaining the selection intensities and total responses gives the breeder the opportunity to implement an unlimited number of selection stages, which would otherwise be very difficult or impossible to do.

Xu and Muir (1991; 1992) indicated that the restriction imposed on the covariance between DCMLGSI values is similar to the Kempthorne and Nordskog (1959) restriction imposed on the expected genetic gain per trait, which makes some traits not change their mean values while the rest of the trait means remain without restrictions (Cerón-Rojas and Crossa, 2018, Chapter 3). Xu and Muir (1992) and Kempthorne and Nordskog (1959) used a projection matrix (*e.g.*, K) to project the LPSI vector of coefficients (*e.g.*, δ) into a space smaller than the original space of δ. The reduction of the space into which the Kempthorne and Nordskog (1959) matrix projects δ is equal to the number of zeros that appears on the expected genetic gain per trait, and the selection response and accuracy decrease as the number of restrictions increases (Cerón-Rojas and Crossa 2018, Chapter 3). However, it is not clear if under the Xu and Muir (1992) restrictions, the selection response and accuracy decrease as the number of stages increases. If this were true, the Xu and Muir (1992) method would not give the breeder the opportunity to implement an unlimited number of stages, because the selection response and accuracy would decrease as the number of stages increases and soon would be null. For example, Xie *et al.* (1997) compared the estimated single-stage LPSI selection response with the estimated DMLPSI selection response for two and three stages and found that at stages 2 and 3, the estimated total DMLPSI selection response explained only 92 and 87%, respectively, of the estimated LPSI selection response. That is, at stage 3, the estimated total DMLPSI selection response was lower (5%) than at stage 2.

### The OCMLGSI and DCMLGSI vector of coefficients

The OCMLGSI is an optimum index and we obtained its vector of coefficients based on the OMLPSI (Young 1964) and LPSI (Smith 1936) theory. For this reason, the OCMLGSI vector of coefficients was easier to obtain than the DCMLGSI vector of coefficients. This last vector is a linear transformation of the OCMLGSI vector of coefficients made by a transforming matrix (K) which is idempotent ($K=K^{2}$) and projects the OCMLGSI vector of coefficients (β) into a space smaller than the original space of β (Cerón-Rojas and Crossa 2018, Chapter 3). Xu and Muir (1992) and Xie and Xu (1998) did not identify that matrix, and for this reason, the equations they used to estimate the decorrelated vector of coefficients seem very complex. This matrix makes the DCMLGSI values independent among stages and is the base for assuming that the DCMLGSI values are normally distributed after stage one. However, due to this matrix, the correlation of the DCMLGSI with the net genetic merit and its selection response after stage one are lower than the correlation of the OCMLGSI with the net genetic merit and its selection response after stage one. Xu and Muir (1992) and Xie and Xu (1998) indicated that the loss of efficiency is justified because their method for obtaining the selection intensities and total responses gives the breeder the opportunity to implement an unlimited number of selection stages, which otherwise would be very difficult or impossible to do. However, Börner and Reinsch (2012) indicated that decorrelated indices should not be used due to the availability of accurate and fast algorithms for exact multidimensional integration to find the selection intensities for the OCMLGSI.

Mi *et al.* (2014) described an R-code algorithm in the multistage context, but it is useful only when the selection is made for a trait at each stage. That is, up to now, there is no quick algorithm for finding the selection intensities for the OCMLGSI for more than two stages, and for this reason, in this work we described a method to find the OCMLGSI selection intensities in the two-stage context.

### The multivariate normality assumption

Based on the normality assumption of the estimated OCMLGSI, DCMLGSI, GEBV, and phenotypic values, we developed and applied the OCMLGSI and DCMLGSI to the real and simulated datasets. The histograms, quantile–quantile plots, and the Shapiro-Wilk and Kolmogorov-Smirnov normality tests of the OCMLGSI and DCMLGSI values at stage two indicated that these values approached the normal distribution. The multivariate normality distribution is very important for breeding plant and animal quantitative traits because these traits show continuous variability and are the result of many gene effects interacting among themselves and with the environment. That is, quantitative traits are the result of unobservable gene effects distributed across plant or animal genomes, which interact among themselves and with the environment to produce the observable characteristic plant and animal phenotypes (Falconer and Mackay 1996). Under the multivariate normal distribution assumption, the indices, the traits, and GEBV can be described using only means, variances, and covariances. In addition, if the traits are not correlated, they are independent. Linear combinations of traits are also normal; and even when the trait phenotypic values do not have normal distribution, this distribution serves as a useful approximation, especially in inferences involving sample mean vectors, which, by the central limit theorem, have multivariate normal distribution (Rencher 2002). By this reasoning, a fundamental assumption in this work was that the net genetic merit and each index have multivariate normal distribution. Under the latter assumption, the regression of the net genetic merit on any linear function of the phenotypic and GEBV values was linear and the total OCMLGSI selection response was the sum of the responses obtained at each stage.

### DCMLGSI and OCMLGSI

The DCMLGSI is an application of the Xie and Xu (1998) index to the genomic selection (GS) context. Based on the LMSI (Lande and Thompson 1990) and on the DMLPSI (Xu and Muir 1992) theoretical results, Xie and Xu (1998) developed their multistage index in the MAS context before Meuwissen *et al.* (2001) GS theory. For this reason, those authors used molecular scores instead of GEBV to predict the net genetic merit at each stage. Because the Xie and Xu (1998) index has the same theoretical and practical problems as the LMSI indicated in the Introduction of this work, we extended the Xie and Xu (1998) index to the GS context and developed the DCMLGSI, which uses GEBV instead of molecular scores in the prediction. This index is free of the problems of the Xie and Xu (1998) index associated with the LMSI. However, because the DCMLGSI is based on the DMLPSI, it has the same advantages and disadvantages as the DMLPSI, indicated in the Introduction of this work.

The OCMLGSI is an application of the OMLPSI (Young 1964; Cerón-Rojas *et al.* 2019 a and b) to the GS context based on the Xie and Xu (1998) idea. The OCMLGSI has the same advantages and disadvantages as the OMLPSI, as indicated in the Introduction of this work, and is an optimum index. In this work, we showed that its selection response and correlation with the net genetic merit is higher than the DCMLGSI selection response and correlation with the net genetic merit.

The OCMLGSI and the DCMLGSI exploit the linkage disequilibrium between markers and QTL that is produced when inbred lines are crossed, which is useful for identifying markers correlated with the traits of interest and for obtaining GEBV (Meuwissen *et al.* 2001). Börner and Reinsch (2012) indicated that GS could replace traditional progeny testing when maximizing the genetic gain per year, as long as the accuracy of GEBV is higher than 0.45. For the simulated dataset, the correlation values between the GEBVs and the traits’ true breeding values in cycle two were 0.52, 0.74, 0.69, and 0.73 for each of the four traits, respectively, whereas in cycle seven, those correlations were 0.40, 0.55, 0.54, and 0.50 for each of the four traits (Cerón-Rojas and Crossa 2019). In all selection cycles, the estimated correlations were higher than, or equal to, 0.45; thus, the GEBVs obtained with the simulated datasets were good predictors of the individual trait breeding values, and the OCMLGSI and the DCMLGSI were good predictors of the net genetic merit because both indices are linear combinations of GEBV at stage 1, and of the GEBV and phenotypic values at stage 2.

The OCMLGSI and the DCMLGSI can only be used in training populations when there is phenotypic and marker information, while the LGSI (Ceron-Rojas *et al.* 2015; Cerón-Rojas and Crossa 2019) is used in testing populations where there is only marker information. However, because both indices incorporate more information than the single-stage LPSI and LGSI, their selection response and correlation with the net genetic merit are higher than the LPSI and the LGSI selection response and correlation with the net genetic merit in all selection cycles. This is the main reason why the OCMLGSI should be used instead of the LPSI and the LGSI.

### Method for obtaining the OCMLGSI selection intensity

The method used in this work to obtain the OCMLGSI selection intensities in a two-stage context is simple and can be programmed in a computer using an R code. Cochran (1951) and Young (1964) described methods to obtain OMLPSI intensities in the two-stage context; however, such methods overestimate the OMLPSI selection intensity (Cerón-Rojas *et al.* 2019 a and b). The method proposed here was good for obtaining the selection intensity values of OCMLGSI in a two-stage context and did not overestimate the OCMLGSI selection intensity. Thus, breeders should use the proposed method when they perform multistage selection.

### The estimated total DCMLGSI selection response was counter-intuitive

The estimated total OCMLGSI and DCMLGSI selection response should be lower, or equal to, the single-stage CLGSI. This implies that when the total proportion selected (*p*) increased, *e.g.*, from 0.05 to 0.30, the estimated total OCMLGSI and DCMLGSI selection response should tend to be more similar to the estimated single-stage CLGSI selection response. This was true for the estimated total OCMLGSI selection response but not true for the estimated total DCMLGSI selection response for the real and simulated datasets. Thus, for the real dataset, when p=0.05, 0.10, 0.20, and 0.30, the estimated total OCMLGSI selection response explained 97.27, 97.35, 97.55, and 97.68%, respectively, of the $\hat{R}$ values, while the estimated total DCMLGSI selection response explained 91.68, 90.64, 89.32, and 88.16%, respectively, of the $\hat{R}$ values. That is, the estimated total DCMLGSI selection response decreased when p values increased. We found similar results for the simulated dataset. Our results were in accordance with those of Börner and Reinsch (2012) and Cerón-Rojas *et al.* (2019b) when these authors compared the optimum with the decorrelated multistage indices in the genomic and phenotypic selection context, respectively. Börner and Reinsch (2012) called the decorrelated multistage index results counter-intuitive and difficult to interpret. Thus, we are in agreement with Börner and Reinsch (2012) that breeders should not use decorrrelated indices when they make multistage selection.

## Conclusion

We evaluated the relative efficiency of two combined multistage linear genomic selection indices. We determined the efficiency of both indices based on the estimated total selection response and correlation of each index with the net genetic merit using real and simulated datasets. In both datasets, we found that the OCMLGSI was a better predictor of the net genetic merit than the DCMLGSI. Therefore, breeders should use the OCMLGSI when performing multistage selection.

## APPENDIX 1

### Genomic breeding values

The *i*^th^ phenotypic value ($y_{i}$) can be denoted as $y_{i}=g_{i}+e_{i}$, where $g_{i}$ is the breeding value (*i.e.*, the average additive effects of the genes an individual receives from both parents) and $e_{i}$ is the residual. It is assumed that $g_{i}$ and $e_{i}$ are independent and that both have normal distribution with an expectation equal to zero and variance $\sigma_{g_{i}}^{2}$ and $\sigma_{e_{i}}^{2}$, respectively. This means that the vector of n observations for trait i (i=1, 2,…,t; t=number of traits) can be written as ${y^{\prime }}_{i}=[\begin{array}{cccc} y_{i1} & y_{i2} & \cdots & y_{in} \end{array}]$, whereas the vector of genomic breeding values associated with vector ${y^{\prime }}_{i}$ (${z^{\prime }}_{i}=[\begin{array}{cccc} z_{i1} & z_{i2} & \cdots & z_{in} \end{array}]$) can be written as

$$z_{i}=Xu_{i},$$ (A1)

where X is an n×m matrix (n= number of observations and m= number of markers in the population) of coded marker values (2−2p, 1−2p, and −2p for genotypes *AA*, *Aa*, and *aa*, respectively) associated with the additive effects of the QTL and $u_{i}$ is a vector with m additive effects of the QTL associated with markers that affect the *i*^th^ trait. It is assumed that $z_{i}$ has multivariate distribution (MVN) with mean **0** and covariance matrix $G\sigma_{z_{i}}^{2}$, where $\sigma_{z_{i}}^{2}$ is the additive genomic variance of $z_{i}$ and $G=XX^{\prime }/c$ is the n×n additive genomic relationship matrix; $c=\sum_{j=1}^{m}2\theta_{j}(1-\theta_{j})$ in an F_2_ population; θ is the frequency of allele *A* and 1−θ is the frequency of allele *a* in the *j*^th^ marker (j=1,2,...,m).

Let $g_{ij}$ (i=1, 2,…,t and j=1,2,...,n) be the *j*^th^ element of the $i^{th}$vector of true genotypic breeding values ${g^{\prime }}_{i}=[\begin{array}{cccc} g_{i1} & g_{i2} & \cdots & g_{in} \end{array}]$; then the covariance between $z_{ij}$ and $g_{ij}$ is $Cov(g_{ij},z_{ij})=\sigma_{z_{i}}^{2}$. That is, the covariance between $z_{ij}$ and $g_{ij}$ is equal to the additive genomic variance of $z_{ij}$ (Dekkers 2007). Let $z^{\prime }=[\begin{array}{cccc} z_{1} & z_{2} & \cdots & z_{t} \end{array}]$ and $g^{\prime }=[\begin{array}{cccc} g_{1} & g_{2} & \cdots & g_{t} \end{array}]$ be the vectors of t traits for the genomic and true breeding values, respectively; then

Γ=Var(z)=Cov(z,g) (A2)

is the covariance matrix of genomic breeding values.

### Estimating marker effects

Let $u^{\prime }=[\begin{array}{cccc} {u^{\prime }}_{1} & {u^{\prime }}_{2} & \cdots & {u^{\prime }}_{t} \end{array}]$ be a vector nt of marker effect values associated with t traits. The *i*^th^ vector ${u^{\prime }}_{i}=[\begin{array}{cccc} u_{i1} & u_{i2} & ... & u_{in} \end{array}]$ (i=1, 2,…,t) of marker effects in the training population can be estimated as ${\hat{u}}_{i}=c^{-1}X^{\prime }{\left[G+\upsilon I_{n}\right]}^{-1}(y_{i}-1\mu_{i})$, where $\upsilon =\frac{\sigma_{e_{i}}^{2}}{\sigma_{g_{i}}^{2}}$, $\sigma_{g_{i}}^{2}$, $\sigma_{e_{i}}^{2}$, and the other parameters were defined earlier. In addition, we can estimate vector $u^{\prime }=[\begin{array}{cccc} {u^{\prime }}_{1} & {u^{\prime }}_{2} & \cdots & {u^{\prime }}_{t} \end{array}]$ in the multi-trait context as

$$\hat{u}=c^{-1}W^{\prime }{\left[\left(I_{t}⊗G)+(N⊗I_{n}\right)\right]}^{-1}(y-\mu ⊗1),$$ (A3)

where $W=I_{t}⊗X$, “⊗” denotes the Kronecker product (Schott 2005); c and X were defined in Equation (A1); $N=RC^{-1}$, R and C are the residual and genotypic covariance matrices for t traits, respectively; $y^{\prime }=[\begin{array}{cccc} {y^{\prime }}_{1} & {y^{\prime }}_{2} & \cdots & {y^{\prime }}_{t} \end{array}]$∼ MVN(μ, V) is a vector of size tn, with covariance matrix $V=C⊗G+R⊗I_{n}$; $I_{t}$ and $I_{n}$ are identity matrices of size t×t and n×n, respectively; $\mu^{\prime }=[\begin{array}{cccc} \mu_{1} & \mu_{2} & \cdots & \mu_{t} \end{array}]$ is a vector t of means, and 1 is a vector of n 1’s.

### Estimating genomic breeding values

We can estimate the vector $z^{\prime }=[\begin{array}{cccc} {z^{\prime }}_{1} & {z^{\prime }}_{2} & \cdots & {z^{\prime }}_{t} \end{array}]$ of genomic breeding values for t traits and n observations as

$$\hat{z}=W\hat{u}.$$ (A4)

Equation (A4) is the vector of GEBV for the multi-trait case.

### Estimating the genomic covariance matrix Γ

Let ${\hat{z}}_{j}=X{\hat{u}}_{j}$ and ${\hat{z}}_{i}=X{\hat{u}}_{i}$ (Equation A4) be the genomic estimated breeding values (GEBV) of $z_{j}=X_{i}u_{j}$ and $z_{i}=Xu_{i}$ (Equation A1), respectively, and denote by ${\hat{\mu }}_{j}$ and ${\hat{\mu }}_{i}$ the arithmetic means of the values of ${\hat{z}}_{j}$ and ${\hat{z}}_{i}$. We can estimate matrix Γ (Equation A2) as

$$\hat{\Gamma }=\{{\hat{\sigma }}_{ji}\},$$ (A5)

where ${\hat{\sigma }}_{ji}=\frac{1}{g}({\hat{z}}_{j}-1{\hat{\mu }}_{j}{)}^{\prime }G_{}^{-1}({\hat{z}}_{i}-1{\hat{\mu }}_{i}{)}^{\prime }$ is the covariance between ${\hat{z}}_{j}=X_{i}{\hat{u}}_{j}$ and ${\hat{z}}_{i}=X{\hat{u}}_{i}$ values (j,i=1, 2, …, t); g is the number of genotypes; 1 is a vector of g 1’s and $G=c^{-1}XX^{\prime }$ is the additive genomic relationship matrix.

### The phenotypic model to estimate the variance components

We estimated matrices P and C using restricted maximum likelihood (REML) because this estimation method does not require a specific design or balanced data and is useful for estimating genetic and residual variance and covariance in any arbitrary pedigree of individuals. In addition, the Expectation and Maximization algorithm allows computing the REML for the variance components (Lynch and Walsh 1998).

Let

$$y_{j}=1\mu_{j}+Zg_{j}+e_{j}$$

be the phenotypic model, where $y_{j}$ is a vector of g (g=number of genotypes in the population) phenotypic values for the $j^{th}$ trait which has multivariate normal distribution (NMV). That is, $y_{j}$ ∼ NMV ($1\mu_{j}$,$V_{j}$), where 1 is a vector of g ones, $\mu_{j}$ is the mean of the $j^{th}$ trait, Z is an identity matrix g×g; $g_{j}$ ∼NMV (**0**, $A\sigma_{g_{j}}^{2}$) is a vector of true breeding values, and $e_{j}$∼NMV(**0**,$I\sigma_{e_{j}}^{2}$) is a vector of residuals. Matrix A denotes the numerical relationship matrix between individuals (Lynch and Walsh 1998), and $V_{j}=A\sigma_{{\text{g}}_{j}}^{2}+I\sigma_{e_{j}}^{2}$. We estimated $\sigma_{{\text{g}}_{j}}^{2}$ and $\sigma_{e_{j}}^{2}$ assuming absence of dominance and epistatic effects.

### Estimating matrices P and C using the Expectation and Maximization algorithm

The Expectation and Maximization algorithm allows computing the REML for the variance components $\sigma_{{\text{g}}_{j}}^{2}$ and $\sigma_{e_{j}}^{2}$ by iterating the following equations:

$$\sigma_{{\text{g}}_{j}}^{2(n+1)}=\sigma_{{\text{g}}_{j}}^{2(n)}+\frac{{\left(\sigma_{{\text{g}}_{j}}^{2(n)}\right)}^{2}}{g}\left[{y^{\prime }}_{j}(T^{\left(n\right)}AT^{\left(n\right)})y_{j}-tr(T^{\left(n\right)}A)\right]$$ (A6)

and

$$\sigma_{e_{j}}^{2(n+1)}=\sigma_{e_{j}}^{2(n)}+\frac{{\left(\sigma_{e_{j}}^{2(n)}\right)}^{2}}{g}\left[{y^{\prime }}_{j}(T^{\left(n\right)}T^{\left(n\right)})y_{j}-tr(T^{\left(n\right)})\right],$$ (A7)

where, after n iterations, $\sigma_{{\text{g}}_{j}}^{2(n+1)}$ and $\sigma_{e_{j}}^{2(n+1)}$ are the estimated variance components of $\sigma_{g_{j}}^{2}$ and $\sigma_{e_{j}}^{2}$, respectively. In Equations (A6) and (A7), tr(.) denotes the trace of the matrices within brackets; $T=V_{j}^{-1}-V_{j}^{-1}1{\left(1^{\prime }V_{j}^{-1}1\right)}^{-1}1^{\prime }V_{j}^{-1}$ and $V_{j}^{-1}$ is the inverse of matrix $V_{j}=A\sigma_{g_{j}}^{2}+I\sigma_{e_{j}}^{2}$. In $T^{\left(n\right)}$, $V_{j}^{-1(n)}$ is the inverse of matrix $V_{j}^{\left(n\right)}=A\sigma_{\gamma_{j}}^{2(n)}+I\sigma_{e_{j}}^{2(n)}$.

The additive genetic and residual covariances between the observations of the $j^{th}$ and *i*^th^ traits, $y_{j}$ and $y_{i}$ ($\sigma_{g_{ij}}$and $\sigma_{e_{ij}}$, j,i=1,2,…,t), can be estimated with REML by adapting Equations (A7) and (A8) as follows. The variance of the sum of $y_{j}$ and $y_{i}$ can be written as $Var(y_{i}+y_{j})=V_{i}+V_{j}+2C_{ij}$, where $V_{i}=A\sigma_{{\text{g}}_{i}}^{2}+I\sigma_{e_{i}}^{2}$ is the variance of $y_{i}$ and $V_{j}=A\sigma_{g_{j}}^{2}+I\sigma_{e_{j}}^{2}$ is the variance of $y_{j}$; in addition, $2C_{ij}=2A\sigma_{g_{ij}}+2I\sigma_{e_{ij}}=2Cov(y_{i},y_{j})$ is the covariance of $y_{j}$ and $y_{i}$, whereas $\sigma_{g_{ij}}$ and $\sigma_{e_{ij}}$ are the additive and residual covariances, respectively, associated with the covariance of $y_{j}$ and $y_{i}$. Thus, one way of estimating $\sigma_{g_{ij}}$ and $\sigma_{e_{ij}}$ is using the following equation:

$$0.5Var(y_{i}+y_{j})-0.5Var(y_{i})-0.5Var(y_{j}),$$ (A8)

for which Equations (A6) and (A7) can be used.

### Estimating the OCMLGSI and DCMLGSI selection response at stages 1 and *2*

By Equations (A5) to (A8), the estimates of matrices P, C, and Γcan be denoted as $\hat{P}$, $\hat{C}$, and $\hat{\Gamma }$, from where the estimated block matrices of Equation (5) can be denoted as $\hat{T}=\left[\begin{array}{cc} \hat{\Gamma } & \hat{\Gamma }\\ \hat{\Gamma } & \hat{P} \end{array}\right]$ and $\hat{\Psi }=\left[\begin{array}{cc} \hat{\Gamma } & \hat{\Gamma }\\ \hat{\Gamma } & \hat{C} \end{array}\right]$ . Thus, at stages 1 and 2, the estimated OCMLGSI selection responses (Equations 2 and 7) are

$${\hat{R}}_{O1}=k_{O1}\sqrt{w^{\prime }\hat{\Gamma }w}\;\text{and}\;{\hat{R}}_{O2}=k_{O2}\sqrt{\hat{\beta^{\prime }}\hat{T}\hat{\beta }},$$ (A9)

where w is the vector of economic weights, and ${\hat{\beta }}^{\prime }=a^{\prime }\hat{\Psi }{\hat{T}}^{-1}$ is an estimate of $\beta \prime =a^{\prime }\Psi T^{-1}$. In a similar manner, the estimated DCMLGSI selection responses at stages 1 and *2* are

$${\hat{R}}_{D1}=k_{D1}\sqrt{w^{\prime }\hat{\Gamma }w}\;\text{and}\;{\hat{R}}_{D2}=k_{D2}\sqrt{\hat{b^{\prime }}\hat{T}\hat{b}},$$ (A10)

where $\hat{b}=\hat{K}\hat{\beta }$ is an estimate of b=Kβ.

## APPENDIX 2

### The OCMLGSI selection intensity for two stages

We describe a method to obtain the OCMLGSI selection intensity for a fixed total proportion $p=q_{1}q_{2}$, where $q_{1}$ and $q_{2}$ are the proportions of individuals selected at stages 1 and 2, respectively. Let $I_{1}={\beta^{\prime }}_{1}x_{1}$ and $I_{2}={\beta^{\prime }}_{2}t$ be the OCMLGSI at stages 1 and 2, respectively, and assume that the indices have bivariate normal distribution. Let $I_{1}$ and $I_{2}$ be transformed as $u_{1}=\frac{I_{1}-\mu_{I_{1}}}{\sigma_{I_{1}}}$ and $u_{2}=\frac{I_{2}-\mu_{I_{2}}}{\sigma_{I_{2}}}$ with mean zero and variance 1.0, where $\mu_{I_{2}}$ and $\mu_{I_{2}}$ are the means, whereas $\sigma_{I_{1}}$ and $\sigma_{I_{2}}$ are the standard deviations of the variance of $I_{1}$ and $I_{2}$, respectively. The selected population has bivariate left truncated normal distribution with probability density function $h(u_{1},u_{2})=\frac{z(u_{1},u_{2})}{p}$, where $p=q_{1}q_{2}$,

$$z(u_{1},u_{2})=\frac{1}{2\pi \sqrt{1-\rho_{12}^{2}}}\exp\left\{-\frac{1}{2(1-\rho_{12}^{2})}\left[u_{1}^{2}+u_{2}^{2}-2\rho_{12}^{}u_{1}^{}u_{2}^{}\right]\right\}$$ (A11)

and $\rho_{12}$ is the correlation between $u_{1}$ and $u_{2}$.

Consider the transformations (Springer 1979, Chapter 3): $v_{1}=u_{1}$ and $v_{2}=\frac{u_{2}-\rho_{12}u_{1}}{\sqrt{1-\rho_{12}^{2}}}$, with Jacobian j, where $j^{-1}=\left|\begin{array}{cc} \frac{\partial v_{1}}{\partial u_{1}} & \frac{\partial v_{1}}{\partial u_{2}}\\ \frac{\partial v_{2}}{\partial u_{1}} & \frac{\partial v_{2}}{\partial u_{2}} \end{array}\right|=\left|\begin{array}{cc} 1 & 0\\ \frac{-\rho_{12}^{}}{\sqrt{1-\rho_{12}^{2}}} & \frac{1}{\sqrt{1-\rho_{12}^{2}}} \end{array}\right|=\frac{1}{\sqrt{1-\rho_{12}^{2}}}$, |∘|denotes the determinant function and ∂ the partial derivatives of $v_{1}$ and $v_{2}$ with respect to $u_{1}$ and $u_{2}$. Thus, $v_{1}^{2}+v_{2}^{2}=\frac{u_{1}^{2}+u_{2}^{2}-2\rho_{12}^{}u_{1}^{}u_{2}^{}}{\left(1-\rho_{12}^{2}\right)}$ and $g(v_{1},v_{2})=\left|j\right|z(u_{1},u_{2})=\left[\frac{1}{\sqrt{2\pi }}e^{-0.5v_{1}^{2}}\right]\left[\frac{1}{\sqrt{2\pi }}e^{-0.5v_{2}^{2}}\right]$. The transformations indicate that variables $v_{1}$ and $v_{2}$ are independent, each with a standard normal distribution.

Variables $v_{1}$ and $v_{2}$ are associated with the truncation points as $u_{1}=v_{1}$ and $u_{2}=v_{2}\sqrt{1-\rho_{12}^{2}}+\rho_{12}v_{1}$. This means that $u_{1}$ and $u_{2}$ values should be obtained in two steps. First, we obtained the values of $v_{1}$ and $v_{2}$ from two independent standard normal distributions; then we obtained the values of $u_{1}=v_{1}$, $u_{2}=v_{2}\sqrt{1-\rho_{12}^{2}}+\rho_{12}v_{1}$, $q_{1}$ and $q_{2}$; finally, we obtained the OCMLGSI selection intensity at stages 1 and 2 as:

$$k_{O1}=\frac{z(u_{1})}{q_{1}}\;\text{and}\;k_{O2}=\frac{z(u_{2})}{q_{2}},$$ (A12)

respectively, where $z(u_{1})=\frac{e^{-0.5u_{1}^{2}}}{\sqrt{2\pi }}$ and $z(u_{2})=\frac{e^{-0.5u_{2}^{2}}}{\sqrt{2\pi }}$ are the height of the ordinate of the normal curve at the lowest values of $u_{1}$ and $u_{2}$ retained, whereas $q_{1}$ and $q_{2}$ are the proportions of the population of animals or plants selected at each stage (Figures 1 and 2). The values of Equation (A12), should maximize the $R_{t}=R_{1}+R_{2}$ value (Figure 3), where $R_{1}=k_{1}\sigma_{I_{1}}$ and $R_{2}=k_{2}\sigma_{I_{2}}$ are the selection responses, whereas $\sigma_{I_{1}}$ and $\sigma_{I_{2}}$ are the standard deviations of the variance of $I_{1}$ and $I_{2}$ at stages 1 and 2, respectively.

## APPENDIX 3

### Adjusting the OCMLGSI covariance matrices at stage two

Matrices $T=\left[\begin{array}{cc} \Gamma & \Gamma \\ \Gamma & P \end{array}\right]$ and $\Psi =\left[\begin{array}{cc} \Gamma & \Gamma \\ \Gamma & C \end{array}\right]$ are affected by prior selection at stage 1, and it is necessary to adjust them to take into consideration the effects that prior selection has on them (Cochran 1951; Cunningham 1975). Because at stage 1 we performed only genomic selection, at stage 2 we adjusted matrices T and Ψ as follows:

$$T^{*}=T-\alpha \frac{\left[\begin{array}{c} \Gamma \\ \Gamma \end{array}\right]ww^{\prime }\left[\begin{array}{cc} \Gamma & \Gamma \end{array}\right]}{w^{\prime }\Gamma w}$$ (A13)

and

$$\Psi^{*}=\Psi -\alpha \frac{\left[\begin{array}{c} \Gamma \\ \Gamma \end{array}\right]ww^{\prime }\left[\begin{array}{cc} \Gamma & \Gamma \end{array}\right]}{w^{\prime }\Gamma w},$$ (A14)

where $T^{*}$and $\Psi^{*}$ are the adjusted matrices, and $\alpha =k_{O1}(k_{O1}-u_{1})$, where $k_{O1}$ and $u_{1}$ are the selection intensity and truncation point, respectively, at stage 1; Γ and w were defined earlier. Thus, the maximized OCMLGSI selection response (Equation 7) and correlation between the OCMLGSI and the net genetic merit (Equation 8) at stage two can be written as $R_{2}=k_{2}\sqrt{\beta^{\prime }T^{*}\beta }$ and $\rho_{HI_{C}}=\frac{\sqrt{\beta^{\prime }T^{*}\beta }}{\sqrt{a^{\prime }\Psi^{*}a}}$. We did not use Equations (A13) and (A14) to adjust matrices T and Ψ for the DCMLGSI because the values between stages of this index are independent.

## References

[bib1] Beyene Y., Semagn K., Mugo S., Tarekegne A., Babu R., 2015 Genetic gains in grain yield through genomic selection in eight bi-parental maize populations under drought stress. Crop Sci. 55: 154–163. 10.2135/cropsci2014.07.0460

[bib2] Börner V., and Reinsch N., 2012 Optimising multistage dairy cattle breeding schemes including genomic selection using decorrelated or optimum selection indices. Genet. Sel. Evol. 44: 1–11. 10.1186/1297-9686-44-1 22252172 PMC3292482

[bib3] Ceron-Rojas J. J., Crossa J., Arief V. N., Basford K., Rutkoski J., Jarquín D., Alvarado G., Beyene Y., Semagn K., and DeLacy I., 2015 A genomic selection index applied to simulated and real data. G3 (Bethesda) 5: 2155–2164. 10.1534/g3.115.019869 PMC4592997 26290571

[bib4] Cerón-Rojas J. J., and Crossa J., 2018 Linear Selection Indices in Modern Plant Breeding, Springer, Cham, the Netherlands, Available at https://link.springer.com/book/10.1007/978–3-319–91223–3, .10.1007/978-3-319-91223-3

[bib5] Cerón-Rojas J. J., Toledo F. H., and Crossa J., 2019 a The relative efficiency of two multi-stage linear phenotypic selection indices to predict the net genetic merit. Crop Sci. 59: 1037–1051. 10.2135/cropsci2018.11.0678 PMC7680945 33343016

[bib6] Cerón-Rojas J. J., Toledo F. H., and Crossa J., 2019 b Optimum and decorrelated constrained multistage linear phenotypic selection indices theory. Crop Sci. 59: 2585–2600. 10.2135/cropsci2019.04.0241 PMC7680945 33343016

[bib7] Cerón-Rojas J. J., and Crossa J., 2019 Efficiency of a constrained linear genomic selection index to predict the net genetic merit in plants. G3 (Bethesda) 9: 3981–3994. 10.1534/g3.119.400677 31570501 PMC6893179

[bib8] Cochran W. G., 1951 Improvement by means of selection Proceedings of the Second Berkeley Symposium on Mathematical Statistics and Probability, 449-470, University of California Press, Berkeley, Calif. Available at https://projecteuclid.org/euclid.bsmsp/1200500247.

[bib9] Crawley M. J., 2015 Statistics: An Introduction Using R, Ed. 2nd John Wiley & Sons Ltd, United Kingdom.

[bib10] Cunningham E. P., 1975 Multi-stage index selection. Theor. Appl. Genet. 46: 55–61. 10.1007/BF00264755 24419606

[bib11] Dekkers J. C. M., 2007 Prediction of response to marker-assisted and genomic selection using selection index theory. J. Anim. Breed. Genet. 124: 331–341. 10.1111/j.1439-0388.2007.00701.x 18076470

[bib12] Falconer D. S., and Mackay T. F. C., 1996 Introduction to Quantitative Genetics, Longman, New York. 10.1093/genetics/167.4.1529 PMC1471025 15342495

[bib13] Hazel L. N., and Lush J. L., 1942 The efficiency of three methods of selection. J. Hered. 33: 393–399. 10.1093/oxfordjournals.jhered.a105102

[bib14] Hazel L. N., 1943 The genetic basis for constructing selection indexes. Genetics 8: 476–490. 10.1093/genetics/28.6.476 PMC1209225 17247099

[bib15] Heffner E. L., Sorrells M. E., and Jannink J. L., 2009 Genomic selection for crop improvement. Crop Sci. 49: 1–12. 10.2135/cropsci2008.08.0512

[bib16] Isik F., Holland J., and Maltecca C., 2017 Genetic Data Analysis for Plant and Animal Breeding, Springer, Cham, the Netherlands 10.1007/978-3-319-55177-7

[bib17] Kabakoff R. I., 2011 R in Action: Data Analysis and Graphics with R, Manning Publications Co., Shelter Island, NY.

[bib18] Kempthorne O., and Nordskog A. W., 1959 Restricted selection indices. Biometrics 15: 10–19. 10.2307/2527598

[bib19] Lynch M., and Walsh B., 1998 Genetics and Analysis of Quantitative Traits, Sinauer, Sunderland, MA.

[bib20] Lande R., and Thompson R., 1990 Efficiency of marker-assisted selection in the improvement of quantitative traits. Genetics 124: 743–756. 1968875 10.1093/genetics/124.3.743 PMC1203965

[bib21] Lange C., and Whittaker J. C., 2001 On prediction of genetic values in marker-assisted selection. Genetics 159: 1375–1381. 11729177 10.1093/genetics/159.3.1375 PMC1461875

[bib22] Meuwissen T. H. E., Hayes B. J., and Goddard M. E., 2001 Prediction of total genetic value using genome-wide dense marker maps. Genetics 157: 1819–1829. 11290733 10.1093/genetics/157.4.1819 PMC1461589

[bib23] Mi X., Utz H. F., Technow F., and Melchinger A. E., 2014 Optimizing resource allocation for multistage selection in plant breeding with R Package. Crop Sci. 54: 1413–1418. 10.2135/cropsci2013.10.0699

[bib24] Mohd-Razali N., and Bee-Wah Y., 2011 Power comparisons of Shapiro-Wilk, Kolmogorov-Smirnov, Lilliefors and Anderson-Darling tests. Journal of Statistical Modeling and Analytics 2: 21–33.

[bib25] Podlich D. W., and Cooper M., 1998 QU-GENE: a simulation platform for quantitative analysis of genetic model. Bioinformatics 14: 632–653. 10.1093/bioinformatics/14.7.632 9730929

[bib26] Rani Das K., and Rahmatullah-Imon A. H. M., 2016 A brief review of tests for normality. Am. J. Theor. Appl. Stat. 5: 5–12. 10.11648/j.ajtas.20160501.12

[bib27] Rencher A. C., 2002 Methods of Multivariate Analysis, Ed. 2nd John Wiley and Son, New York 10.1002/0471271357

[bib28] Saxton A. M., 1983 A comparison of exact and sequential methods in multi-stage index selection. Theor. Appl. Genet. 66: 23–28. 10.1007/BF00281843 24263627

[bib29] Shapiro S. S., and Wilk M. B., 1965 An analysis of variance test for normality (complete samples). Biometrika 52: 591–611. 10.1093/biomet/52.3-4.591

[bib30] Schott J. R., 2005 Matrix Analysis for Statistics, Ed. 2nd John Wiley & Sons, Inc., Hoboken, New Jersey.

[bib31] Smith F. H., 1936 A discriminant function for plant selection. Ann. Eugen. 7: 240–250. 10.1111/j.1469-1809.1936.tb02143.x

[bib32] Springer M. D., 1979 The Algebra of Random Variables, Wiley, New York.

[bib33] Xie C., Xu S., and Mosjidis J. A., 1997 Multistage selection indices for maximum genetic gain and economic efficiency in red clover. Euphytica 98: 75–82. 10.1023/A:1003074814916

[bib34] Xie C., and Xu S., 1998 Efficiency of multistage marker-assisted selection in the improvement of multiple quantitative traits. Heredity 80: 489–498. 10.1046/j.1365-2540.1998.00308.x 9618913

[bib35] Xu S., and Muir W. M., 1991 Multistage selection for genetic gain by orthogonal transformation. Genetics 129: 963–974. 1752432 10.1093/genetics/129.3.963 PMC1204762

[bib36] Xu S., and Muir W. M., 1992 Selection index updating. Theor. Appl. Genet. 83: 451–458. 10.1007/BF00226533 24202591

[bib37] Xu S., Martin T. G., and Muir W. M., 1995 Multistage selection for maximum economic return with an application to beef cattle breeding. J. Anim. Sci. 73: 699–710. 10.2527/1995.733699x 7608002

[bib38] Young S. S. Y., 1964 Multi-stage selection for genetic gain. Heredity 19: 131–145. 10.1038/hdy.1964.11

